# Expanding the Diversity of Mycobacteriophages: Insights into Genome Architecture and Evolution

**DOI:** 10.1371/journal.pone.0016329

**Published:** 2011-01-27

**Authors:** Welkin H. Pope, Deborah Jacobs-Sera, Daniel A. Russell, Craig L. Peebles, Zein Al-Atrache, Turi A. Alcoser, Lisa M. Alexander, Matthew B. Alfano, Samantha T. Alford, Nichols E. Amy, Marie D. Anderson, Alexander G. Anderson, Andrew A. S. Ang, Manuel Ares, Amanda J. Barber, Lucia P. Barker, Jonathan M. Barrett, William D. Barshop, Cynthia M. Bauerle, Ian M. Bayles, Katherine L. Belfield, Aaron A. Best, Agustin Borjon, Charles A. Bowman, Christine A. Boyer, Kevin W. Bradley, Victoria A. Bradley, Lauren N. Broadway, Keshav Budwal, Kayla N. Busby, Ian W. Campbell, Anne M. Campbell, Alyssa Carey, Steven M. Caruso, Rebekah D. Chew, Chelsea L. Cockburn, Lianne B. Cohen, Jeffrey M. Corajod, Steven G. Cresawn, Kimberly R. Davis, Lisa Deng, Dee R. Denver, Breyon R. Dixon, Sahrish Ekram, Sarah C. R. Elgin, Angela E. Engelsen, Belle E. V. English, Marcella L. Erb, Crystal Estrada, Laura Z. Filliger, Ann M. Findley, Lauren Forbes, Mark H. Forsyth, Tyler M. Fox, Melissa J. Fritz, Roberto Garcia, Zindzi D. George, Anne E. Georges, Christopher R. Gissendanner, Shannon Goff, Rebecca Goldstein, Kobie C. Gordon, Russell D. Green, Stephanie L. Guerra, Krysta R. Guiney-Olsen, Bridget G. Guiza, Leila Haghighat, Garrett V. Hagopian, Catherine J. Harmon, Jeremy S. Harmson, Grant A. Hartzog, Samuel E. Harvey, Siping He, Kevin J. He, Kaitlin E. Healy, Ellen R. Higinbotham, Erin N. Hildebrandt, Jason H. Ho, Gina M. Hogan, Victoria G. Hohenstein, Nathan A. Holz, Vincent J. Huang, Ericka L. Hufford, Peter M. Hynes, Arrykka S. Jackson, Erica C. Jansen, Jonathan Jarvik, Paul G. Jasinto, Tuajuanda C. Jordan, Tomas Kasza, Murray A. Katelyn, Jessica S. Kelsey, Larisa A. Kerrigan, Daryl Khaw, Junghee Kim, Justin Z. Knutter, Ching-Chung Ko, Gail V. Larkin, Jennifer R. Laroche, Asma Latif, Kohana D. Leuba, Sequoia I. Leuba, Lynn O. Lewis, Kathryn E. Loesser-Casey, Courtney A. Long, A. Javier Lopez, Nicholas Lowery, Tina Q. Lu, Victor Mac, Isaac R. Masters, Jazmyn J. McCloud, Molly J. McDonough, Andrew J. Medenbach, Anjali Menon, Rachel Miller, Brandon K. Morgan, Patrick C. Ng, Elvis Nguyen, Katrina T. Nguyen, Emilie T. Nguyen, Kaylee M. Nicholson, Lindsay A. Parnell, Caitlin E. Peirce, Allison M. Perz, Luke J. Peterson, Rachel E. Pferdehirt, Seegren V. Philip, Kit Pogliano, Joe Pogliano, Tamsen Polley, Erica J. Puopolo, Hannah S. Rabinowitz, Michael J. Resiss, Corwin N. Rhyan, Yetta M. Robinson, Lauren L. Rodriguez, Andrew C. Rose, Jeffrey D. Rubin, Jessica A. Ruby, Margaret S. Saha, James W. Sandoz, Judith Savitskaya, Dale J. Schipper, Christine E. Schnitzler, Amanda R. Schott, J. Bradley Segal, Christopher D. Shaffer, Kathryn E. Sheldon, Erica M. Shepard, Jonathan W. Shepardson, Madav K. Shroff, Jessica M. Simmons, Erika F. Simms, Brandy M. Simpson, Kathryn M. Sinclair, Robert L. Sjoholm, Ingrid J. Slette, Blaire C. Spaulding, Clark L. Straub, Joseph Stukey, Trevor Sughrue, Tin-Yun Tang, Lyons M. Tatyana, Stephen B. Taylor, Barbara J. Taylor, Louise M. Temple, Jasper V. Thompson, Michael P. Tokarz, Stephanie E. Trapani, Alexander P. Troum, Jonathan Tsay, Anthony T. Tubbs, Jillian M. Walton, Danielle H. Wang, Hannah Wang, John R. Warner, Emilie G. Weisser, Samantha C. Wendler, Kathleen A. Weston-Hafer, Hilary M. Whelan, Kurt E. Williamson, Angelica N. Willis, Hannah S. Wirtshafter, Theresa W. Wong, Phillip Wu, Yun jeong Yang, Brandon C. Yee, David A. Zaidins, Bo Zhang, Melina Y. Zúniga, Roger W. Hendrix, Graham F. Hatfull

**Affiliations:** 1 Department of Biological Sciences, Carnegie Mellon University, Pittsburgh, Pennsylvania, United States of America; 2 Biology Department, College of William & Mary, Williamsburg, Virginia, United States of America; 3 Biology Department, A. Paul Schaap Science Center, Hope College, Holland, Michigan, United States of America; 4 Howard Hughes Medical Institute, Science Education Alliance, Chevy Chase, Maryland United States of America; 5 Department of Biology, James Madison University, Harrisonburg, Virginia, United States of America; 6 Department of Zoology, Oregon State University, Corvallis, Oregon, United States of America; 7 Department of Biology, Spelman College, Atlanta, Georgia, United States of America; 8 Division of Biological Sciences, University of California San Diego, La Jolla, California, United States of America; 9 Biological Sciences, University of California Santa Cruz, Santa Cruz, California, United States of America; 10 Department of Biology, University of Louisiana at Monroe, Monroe, Louisiana, United States of America; 11 Department of Biological Sciences, University of Mary Washington, Fredericksburg, Virginia, United States of America; 12 Department of Biological Sciences, University of Maryland, Baltimore, Maryland, United States of America; 13 Department of Biological Sciences, University of Pittsburgh, Pittsburgh, Pennsylvania, United States of America; 14 Department of Biology, Washington University in St. Louis, St. Louis, Missouri, United States of America; Cairo University, Egypt

## Abstract

Mycobacteriophages are viruses that infect mycobacterial hosts such as *Mycobacterium smegmatis* and *Mycobacterium tuberculosis*. All mycobacteriophages characterized to date are dsDNA tailed phages, and have either siphoviral or myoviral morphotypes. However, their genetic diversity is considerable, and although sixty-two genomes have been sequenced and comparatively analyzed, these likely represent only a small portion of the diversity of the mycobacteriophage population at large. Here we report the isolation, sequencing and comparative genomic analysis of 18 new mycobacteriophages isolated from geographically distinct locations within the United States. Although no clear correlation between location and genome type can be discerned, these genomes expand our knowledge of mycobacteriophage diversity and enhance our understanding of the roles of mobile elements in viral evolution. Expansion of the number of mycobacteriophages grouped within Cluster A provides insights into the basis of immune specificity in these temperate phages, and we also describe a novel example of apparent immunity theft. The isolation and genomic analysis of bacteriophages by freshman college students provides an example of an authentic research experience for novice scientists.

## Introduction

Bacteriophages are the most numerous biological entities in the biosphere, with an estimated 10^31^ particles [1]. The global population is highly dynamic with an estimated 10^23^ phage infections per second [2], and has likely been evolving for perhaps two to four billion years. Not surprisingly, this has given rise to a genetically highly diverse population [3], [4]. Most bacteriophages do not extend their host range beyond a single bacterial genus, and host specificity likely offers a substantial impediment to the free exchange of genetic material between phages of different bacterial hosts [5]. Consequently, it is unusual to find extensive nucleotide sequence similarity among phages of different hosts; such phages often share few if any genes identifiable through amino acid sequence comparisons.

Remarkably, phages capable of infecting a single bacterial species can also be highly diverse, as are for example the genetically distinct DNA phages of *Escherichia coli*, such as φX174, M13, lambda, T1, T4, T5, and T7 [6]. This is further exemplified with the mycobacteriophages – viruses infecting mycobacterial hosts – of which sixty-two genomes of phages known to infect *Mycobacterium smegmatis* mc^2^155 have been sequenced [7], [8], [9]. All of these are dsDNA tailed phages, restricted to two morphotypes, the Siphoviridae and the Myoviridae [10]. When grouped according to gross nucleotide sequence comparisons, nine major clusters emerge (A–I), with five genomes (Giles, Corndog, Wildcat, Omega, TM4) being singletons with no close relatives [7]. Five of the clusters are quite diverse and can be divided into subclusters, such that there are approximately 21 distinct genome types [7]. Only two of these (Subclusters C1 and C2) correspond to phages with myoviral morphologies (with contractile tails), illustrating the high genetic repertoire of those with siphoviral morphotypes (long non-contractile tails).

As with other groups of bacteriophages – including those infecting *Burkholderia* [11], *Pseudomonas* [12], *Salmonella* [13], or *Staphylococcus* [14] – a high proportion (∼80%) of the predicted mycobacteriophage protein-coding genes are novel in the sense of not having detectable homologues in the public databases [7]. The genomes also have characteristic mosaic architectures, such that each individual genome can be considered to be composed of a series of individual modules, each of which may be shared by genomes that otherwise may not be closely related [15], [16]. In the mycobacteriophages it is common for these individual modules to correspond to single genes, and this mosaicism can be presented by assorting related genes (through shared amino acid sequences) into phamilies (phams), representing phylogenies of these phams using phamily circles [7], [16].

Bacteriophage genome mosaicism as revealed by comparative analyses can be generated by a variety of mechanisms. For example, it is not uncommon to find morons, segments of DNA present in one genome but absent from a related genome, which typically contain an open reading frame flanked by a promoter and a terminator [3], [17]. Insertions and rearrangements can also arise by the action of transposons [8] and the action of other mobile elements such as introns [18], inteins [19] and those coding for homing endonucleases [20], all of which are observed. Many phages encode conservative site-specific recombination systems such as integrases and DNA-invertases, which also mediate DNA rearrangements. Junctions between mosaic modules could be generated by homologous recombination at short conserved boundary sequences [21], [22], but because such sequences cannot be identified at most mosaic boundaries, illegitimate recombination events independent of extensive sequence homology represent an attractive mechanism for generating mosaicism [23], [24]. Phage-encoded recombinases may facilitate such events [25].

Here we describe the sequence determination of eighteen new mycobacteriophage genomes isolated from geographically dispersed locations across the United States. The majority of these phages were isolated, sequenced, and annotated by freshman students in a structured and integrated education and research program supported by the Howard Hughes Medical Institute (HHMI) Science Education Alliance (SEA). Genomic comparison with previously described mycobacteriophage genomes reveals many new insights into mycobacteriophage diversity, evolution, and biological functions. First, we do not see any close correlation between genome type and geographical location or time of isolation. Second, it is evident that the mycobacteriophage population at-large remains under-sampled, because new singleton phages with genomes entirely unrelated to known phages – as well as new relatives of previously classified singleton genomes – can still be isolated. Third, the newly sequenced genomes provide insights into the mechanisms for genome variability including mycobacteriophage mobile elements (MPME), homing endonucleases and inteins. Lastly, we describe new insights into the basis of superinfection immunity among the Cluster A mycobacteriophages, and identify an unusual example of immunity theft.

## Results and Discussion

### 1. Isolation, clustering, and demography of newly isolated mycobacteriophages

#### Isolation and genomic characterization of new mycobacteriophages

Thirteen new mycobacteriophages were isolated at 12 different institutions in the autumn of 2008 as part of a freshman research course in phage discovery and genomics administered by the Howard Hughes Medical Institute (HHMI) Science Education Alliance (SEA) [26] (Table 1). An additional five mycobacteriophages were isolated and characterized within the Phage Hunters Integrating Research and Education (PHIRE) program at the University of Pittsburgh [16] (Table 1). All of these mycobacteriophages were isolated from environmental samples as described previously [7], [16] using *M. smegmatis* mc^2^155 [27] as a host. Seven of the phages were isolated by direct plating of samples on *M. smegmatis* lawns (ET08, Fang, Phlyer, Puhltonio, RedRock, Scoot17C, SkiPole), and eleven were recovered after enrichment in liquid cultures of *M. smegmatis* (Angelica, Colbert, CrimD, Island3, Eagle, Hope, LeBron, LRRHood, Peaches, Pumpkin, and, UncleHowie). The isolation of Puhltonio and description of some of its characteristics has been reported recently [26].

**Table 1:** New mycobacteriophages described in this study.

Phage	Size (bp)	GC (%)	ORFs	tRNA (#)	tmRNA (#)	Ends	Accession	Cluster	Origins	Finder#
Angelica	59598	66.4	94	1	0	11-base 3′	HM152764	K1	Clayton MO	Washington Univ. (SEA)
Colbert	67774	66.5	100	0	0	Circ Perm	GQ303259.1	B1	Corvallis OR	Oregon State (SEA)
CrimD	59798	66.5	95	1	0	11-base 3′	HM152767	K1	Williamsburg VA	Wm & Mary (SEA)
Eagle	51436	63.4	87	0	0	10-base 3′	HM152766	A4	Fredericksburg VA	Mary Washington (SEA)
ET08	155445	64.6	218	30	1	Circ Perm	GQ303260.1	C1	La Jolla CA	UCSD (SEA)
Hope	41901	66.6	63	0	0	11-base 3′	GQ303261.1	G	Lithonia GA	Spelman College (SEA)
Island3	47287	66.8	76	0	0	11-base 3′	HM152765	I1	Pittsburgh PA	CMU (SEA)
LeBron	73453	58.8	123	9	0	10-base 3′	HM152763	Sin	Allensville NC	JMU (SEA)
LRRHood	154349	64.7	224	30	1	Circ Perm	GQ303262.1	C1	Santa Cruz CA	UCSC (SEA)
Peaches	51376	63.9	86	0	0	10-base 3′	GQ303263.1	A4	Monroe, LA	UL Monroe (SEA)
Puhltonio	68323	66.4	97	0	0	Circ Perm	GQ303264.1	B1	Catonsville MD	UMBC (SEA)
Pumpkin	74491	63.0	143	2	0	9-base 3′	GQ303265.1	E	Holland MI	Hope College (SEA)
UncleHowie	68016	66.5	98	0	0	Circ Perm	GQ303266.1	B1	University City MO	Washington Univ. (SEA)
Fang	68569	66.5	102	0	0	Circ Perm	GU247133	B1	O'Hara Twp PA	S. Leuba (PHIRE)
Phlyer	69378	67.5	103	0	0	Circ Perm	FJ641182.1	B3	Pittsburgh PA	D. Altman (PHIRE)
RedRock	53332	64.5	95	1	0	10-base 3′	GU339467	A2	Sedona AZ	D. Jacobs-Sera
Scoot17C	68432	66.5	102	0	0	Circ Perm	GU247134	B1	Pittsburgh PA	V. Hohenstein (PHIRE)
Skipole	53137	62.7	102	0	0	10-base 3′	GU247132	A1	Champlin Park MN	S. Glennon (PHIRE)

# Finder is shown as either a participating institution in the Science Education Alliance (SEA), or a student in the Phage Hunters Integrating Research and Education (PHIRE) program at the University of Pittsburgh.

Nucleic acids were isolated from each of the 18 phages and all were shown to have dsDNA genomes. Complete genome sequences were determined either by shotgun Sanger sequencing or by 454 pyrosequencing. Sequence ambiguities and genome ends were resolved by direct sequencing of genomic DNA templates using oligonucleotide primers. Eight of the phages were shown to have circularly permuted genomes, and for annotation purposes the left end was defined as either codon one of the large terminase gene, or such as to correspond to the left end of closely related genomes (Table 1). Ten of the genomes have defined termini with 3′ single strand extensions of 9, 10, or 11 bases in length (Table 1). Genome lengths vary from 41,901 bp (Hope) to 155,445 bp (ET08) (Table 1). Comparative analysis of the eighteen newly isolated mycobacteriophage genomes was carried out using the 60 mycobacteriophages analyzed previously [7] as well as mycobacteriophages Angel [8] and Ardmore [9].

All of the newly sequenced genomes were annotated for open reading frames (orfs), tRNA genes, and other features. All of the predicted protein-coding genes – together with those of the 62 previously annotated genomes – were used to assemble a database in the program Phamerator (S.G.C., R.W.H, and G.F.H, unpublished), which compares each of the predicted amino acid sequences with all others and sorts the genes into phams [16]. Using threshold criteria of 32.5% identity with ClustalW and a BlastP E-value of 10^−50^, the 9,014 predicted genes assembled into 2,345 phams, of which 1,108 (47.2%) are orphams (phams with only a single gene member). The largest pham contains 104 members, and the mean pham size is 3.84 (median is 2.0). The pham assignments are shown in Table S2.

#### Virion morphologies

Each of the 18 newly isolated mycobacteriophages was examined by electron microscopy (data not shown). Sixteen of these have siphoviral morphologies—long flexible non-contractile tails—and two (ET08 and LRRHood) have myoviral morphologies—shorter contractile tails; all have isometric icosahedral heads, with the exception of Island3, which is prolate (data not shown). The length∶width ratio of the Island3 capsid is 2.5∶1, which is similar to those measured for mycobacteriophages of Cluster I, Brujita and Che9c [7], and the unsequenced phage R1 [28].

#### Classification of newly isolated mycobacteriophages into clusters and subclusters

**Figure 1:**
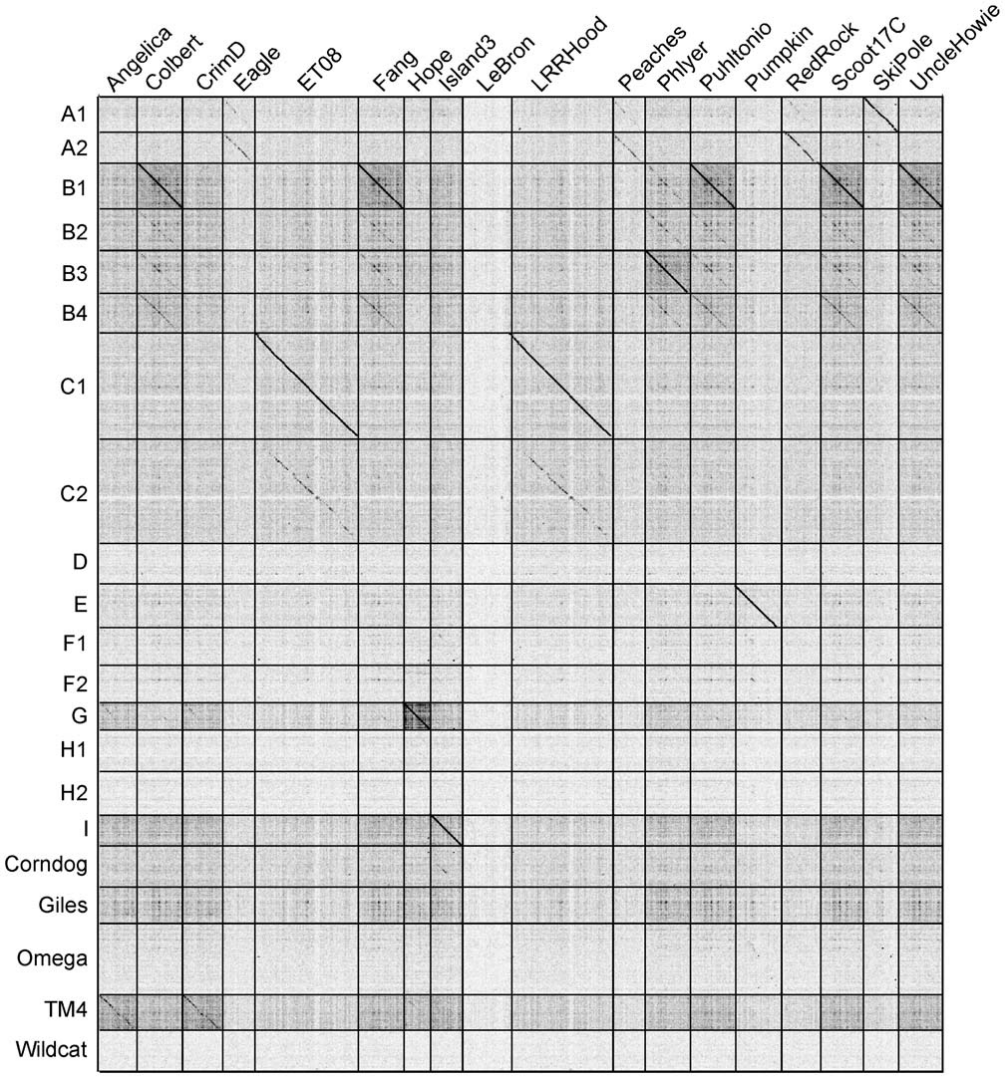
Nucleotide sequence comparison of 18 newly isolated mycobacteriophage genomes. A concatenated file of all 18 newly sequenced genomes (horizontal axis) was compared against a concatenated file of a representative genome of each of the clusters, subclusters, and singleton genomes [as defined in [7]] (vertical axis) using Gepard [29]. The representative genomes on the vertical axis are: A1 (Bethlehem), A2 (D29), B1 (Chah), B2 (Qyrzula), B3 (Phaedrus), B4 (Cooper), C1 (Bxz1), C2 (Myrna), D (Adjutor), E (244), F1 (Boomer), F2 (Che9d), G (Angel), H1 (Konstantine), H2 (Barnyard), I (Brujita), singletons shown are Corndog, Giles, Omega, TM4, and Wildcat.

Each of the newly sequenced genomes was compared with known mycobacteriophage genomes using the dotplot program Gepard [29] (Fig. 1). A variety of relationships are observed, and 15 of the 18 can be assigned readily to one of the previously designated clusters (Fig. 1, Table 2), using the previously stated criteria that cluster membership is warranted if there is nucleotide sequence similarity recognizable in the dotplot and spanning more than half of the genome lengths [7]. Six of the phages clearly belong to Cluster B with five (Colbert, Fang, Puhltonio, Scoot17C and UncleHowie) in the B1 subcluster and one (Phlyer) in B3 (Fig. 1); no new members of Subclusters B2 or B4 were identified (Table 2). ET08 and LRRHood are members of the C1 subcluster, Pumpkin is a new member of E cluster, and Hope is a Cluster G phage (Fig. 1, Table 2). Genome maps of the newly sequenced phages are shown in Fig. S1.

**Table 2:** Assignment of mycobacteriophage genomes into clusters and subclusters.

A	B	C	D	E	F	G	H	I	K	Sin
**A1**	**B1**	**C1**			**F1**		**H1**	**I1**	**K1**	
Bxb1	Chah	Bxz1	PBI1	Cjw1	Che8	Halo	Predator	Brujita	***Angelica***	Corndog
Bethlehem	Orion	Catera	Plot	244	PMC	BPs	Konstantine	***Island3***	***CrimD***	Omega
U2	PG1	Cali	Adjutor	Kostya	Llij	Angel				Wildcat
DD5	***Colbert***	Rizal	Butterscotch	Porky	Boomer	***Hope***	**H2**	**I2**	**K2**	Giles
Jasper	***Fang***	ScottMcG	Gumball	***Pumpkin***	Fruitloop		Barnyard	Che9c	TM4	***LeBron***
KBG	***Puhltonio***	Spud	Troll4		Pacc40					
Lockley	***Scoot17c***	***ET08***			Ramsey					
Solon	***UncleHowie***	***LRRHood***			Tweety					
***SkiPole***					Ardmore					
	**B2**	**C2**								
**A2**	Rosebush	Myrna			**F2**					
D29	Qyrzula				Che9d					
L5										
Che12	**B3**									
Pukovnik	Pipefish									
***RedRock***	Phaedrus									
	***Phlyer***									
**A3**										
Bxz2	**B4**									
	Cooper									
**A4**	Nigel									
***Eagle***										
***Peaches***										

Newly sequenced genomes are shown in bold italic type. Sin: Singletons.

Phages Eagle, Peaches, SkiPole and RedRock are all members of Cluster A as evident by nucleotide sequence comparison (Fig. 1), but their relationships with other Cluster A phages suggest a revision of the number of subclusters to create new Subclusters A3 and A4 (Table 3). Details of this analysis are given in Figure S2.

**Figure 2:**
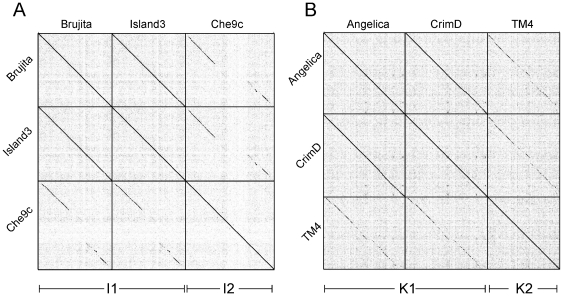
Nucleotide comparison of Cluster I and Cluster K genomes. The three genomes of Cluster I (**A**) and the three genomes of Cluster K (**B**) were concatenated and compared against themselves using Gepard [29]. Each Cluster is subdivided into Subclusters (I1 and I2, and K1 and K2 respectively), as shown below the dotplots.

Island3 has close similarity with Cluster I phages (Fig. 1, Fig. 2A), which as we noted above, also share similar prolate capsid morphologies. Previously, there were two members of Cluster I, Brujita and Che9c, although these are quite distantly related (75.7% ANI; Table 4) [7]. Island3 is, however, a very close relative of Brujita (99.8% ANI; Fig. 2A, Table 4) and we propose that Brujita and Island3 form Subcluster I1, whereas Che9c is currently the sole member of Subcluster I2 (Fig. 2A, Table 4).

**Table 3:** Division of Cluster A into Subclusters A1, A2, A3, and A4.

	Beth	Bxb1	DD5	J'per	KBG	L'ley	U2	S'on	*S'Pole*	Che12	D29	L5	P'vnik	*R'ock*	Bxz2	*Eagle*	*P'hes*
Bethlehem	1	0.89	0.93	0.91	0.94	0.92	0.94	0.94	0.92	0.62	0.62	0.61	0.61	0.61	0.63	0.63	0.62
Bxb1		1	0.88	0.86	0.89	0.89	0.90	0.91	0.87	0.64	0.63	0.63	0.63	0.62	0.65	0.64	0.63
DD5			1	0.92	0.92	0.99	0.92	0.93	0.93	0.61	0.61	0.61	0.62	0.61	0.63	0.63	0.62
Jasper				1	0.89	0.92	0.90	0.91	0.91	0.62	0.61	0.61	0.62	0.61	0.67	0.62	0.62
KBG					1	0.91	0.93	0.93	0.91	0.62	0.61	0.61	0.62	0.61	0.64	0.63	0.63
Lockley						1	0.92	0.93	0.93	0.62	0.61	0.61	0.62	0.61	0.63	0.63	0.62
U2							1	0.93	0.91	0.62	0.62	0.62	0.62	0.61	0.63	0.63	0.63
Solon								1	0.92	0.62	0.62	0.62	0.62	0.61	0.63	0.63	0.63
***SkiPole***									1	0.62	0.62	0.62	0.62	0.62	0.63	0.63	0.63
Che12										1	0.79	0.81	0.75	0.75	0.67	0.68	0.68
D29											1	0.80	0.76	0.76	0.69	0.69	0.68
L5												1	0.76	0.76	0.68	0.66	0.66
Pukovnik													1	0.76	0.67	0.69	0.68
***RedRock***														1	0.68	0.66	0.67
Bxz2															1	0.70	0.70
***Eagle***																1	0.97
***Peaches***																	1

Values show Average Nucleotide Identities. Newly isolated phage names are shown in bold italic type. Bethlehem, Bxb1, DD5, Jasper, KBG, Lockley, U2, Solon and SkiPole constitute Subcluster A1. Che12, D29, L5, Pukovnik, and RedRock constitute Subcluster A2. Bxz2 is the sole member of Subcluster A3, and Eagle and Peaches form Subcluster A4.

**Table 4:** Division of Cluster I into subclusters.

	Brujita	*Island3*	Che9c
Brujita	1	0.998	0.757
***Island3***		1	0.755
Che9c			1

Values show Average Nucleotide Identities. Newly isolated phage name is shown in bold italic type. Brujita and Island3 constitute Subcluster I1, and Che9c forms Subcluster I2.

TM4 was previously identified as a singleton phage with, by definition, no other closely related genomes. However, two of the new phages described here – Angelica and CrimD –have evident sequence similarity to TM4 (Fig. 1, Fig. 2B). Angelica and CrimD are quite closely related to each other (93.5% ANI; Table 5), so we propose that these two constitute Subcluster K1, and that the more distant TM4 forms Subcluster K2 (Fig. 2B, Table 5).

**Table 5:** Division of Cluster K into subclusters.

	*Angelica*	*CrimD*	TM4
***Angelica***	1	0.935	0.689
***CrimD***		1	0.692
TM4			1

Values show Average Nucleotide Identities. Newly isolated phage names are shown in bold italic type. Angelica and CrimD constitute Subcluster K1, and TM4 forms Subcluster K2.

**Figure 3:**
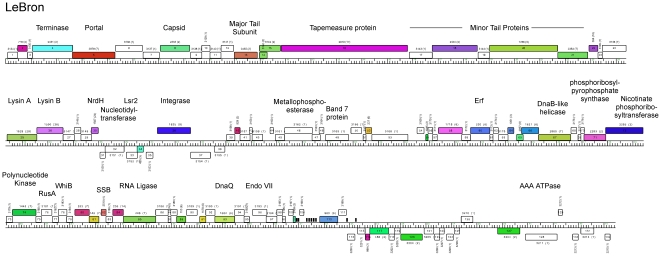
Genome organization of mycobacteriophage LeBron. The LeBron genome is represented as a horizontal bar with markers, and the 132 predicted ORFs are shown as colored boxes either above (rightwards transcribed) or below (leftwards transcribed) the genome. Gene names are shown inside the boxes and the phams to which they belong are indicated above, with the total number of pham members shown in parentheses; white ORFs are orphams with, by definition, no close mycobacteriophage homologues. tRNA genes are shown as short black bars. Putative gene functions primarily identified through database searches are shown.

LeBron is a singleton phage and thus presents a new genome type not closely related at the nucleotide level to any previously sequenced mycobacteriophages (Fig. 1). It has a 73,453 bp genome and contains nine tRNA genes and 123 predicted open reading frames; 80 of these (65%) are orphams, not closely related to other known mycobacteriophage genes (Fig. 3). We suspect LeBron is a temperate phage because it encodes a tyrosine-integrase (gp36), although no homologues of known phage repressors are evident. LeBron is unusual in that an *attP* site cannot be readily predicted from bioinformatic analysis. BlastN searches of regions close to the integrase gene identified no segments of nucleotide similarity to tRNA genes within the *M. smegmatis* chromosome. A number of smaller segments of sequence similarity (<20 bp) were identified, one of which may correspond to an *attP* common core, presumably reflecting integration into a non-tRNA *attB* site. LeBron encodes a number of genes of interest including two predicted Holliday-junction enzymes, one related to RusA (gp 76) and one an endonuclease VII-like protein (gp96) (Fig. 3).

Among the LeBron putative virion structure and assembly genes, eight (gp4, gp5, gp8, gp13, gp14, gp15, gp16, gp18) are most closely related to predicted structural proteins of Wildcat (also a singleton phage). These include the major capsid subunit (gp8) and major tail subunit (gp13) proteins, which share a common ∼70 aa C-terminal extension, a property described previously for proteins of analogous function in both Bxb1 [30] and Wildcat [7]. In both LeBron and Wildcat these extensions are related to Ig-like domains which have been reported as common features of other viruses [31]. The functional roles of these mycobacteriophage extensions are unknown, but a suggestion has been made that they may have a role in the initial stages of the phage's adsorption to a cell [31]. We note that in total there are likely to be >750 copies of these Ig-like domains on each virion particle, 415 in the capsid and the remainder in the tail, indicating that they must be a dominant feature of the virion surface.

#### Geographic and temporal distribution of newly isolated mycobacteriophages

**Figure 4:**
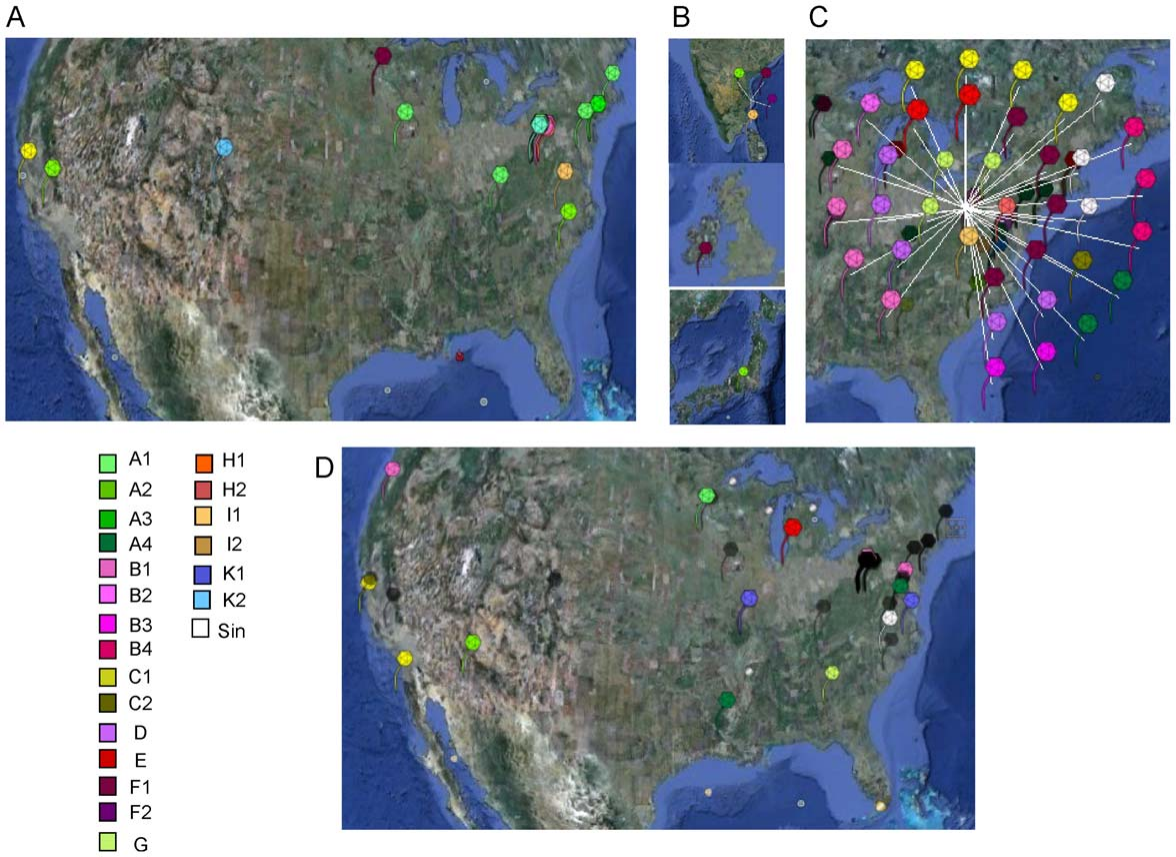
Geographical distributions of genomically characterized mycobacteriophages. **A–C**. Geographical distribution of the isolation sites of 60 previously described sequenced mycobacteriophages according to cluster assignation: United States (**A**), India, Ireland, and Japan (**B**), and Pittsburgh, PA (**C**). Locations of newly isolated mycobacteriophages reported in this study colored according to cluster; locations of previously isolated genomes are shown in black (**D**).

As the number of sequenced mycobacteriophage genomes has expanded over time, the collection has grown in unpredictable patterns. The majority (39 of 62) of previously described and genomically characterized mycobacteriophages were isolated in Pennsylvania, USA, but the distributed phage discovery education program implemented by the HHMI Science Education Alliance provides a geographically broad basis for isolating new phages, raising the question as to whether there are geographical constraints to the types of phages that are present and can be isolated with the commonly used procedures. The locations of the new phages' isolation are shown in Figure 4D. We find no strong correlation between the cluster types of newly isolated phages and their discovery locations, but there are nonetheless hints that the distribution may not be completely random. For example, the F1 subcluster contains a substantial proportion of the 80 genomes characterized (11%) but all except for one (Ardmore, isolated in Ireland) were isolated previously in the Pittsburgh, PA region; none of the 18 newly identified genomes are in the F1 subcluster. In addition, we note that only one new A1 genome – SkiPole, from Minneapolis – was isolated outside of Pennsylvania. The number of genomes is still small, and it remains to be seen whether these potential geographical preferences for certain phage types are observed with a larger sampling of mycobacteriophages.

An alternative explanation for non-random recovery of phage types is that there are temporal patterns of phage prevalence. We have therefore also examined the emergence and accumulation of mycobacteriophage isolates for which complete genome sequences have been determined (Fig. S3). While again there are no strong patterns, there are some hints of non-randomness, but substantially more data will be required before such indications of patterns rise to the level of statistical significance.

### 2. New insights into mycobacteriophage evolution and function

#### New integration specificities: CrimD and Angelica integrate into the host tmRNA gene

Nine of the newly sequenced phages encode integrases, of which two are serine-recombinases (Peaches and SkiPole), and seven are tyrosine-recombinases (Angelica, CrimD, Eagle, Hope, Island3, LeBron, Pumpkin). Because of the small common core sequences used by serine-integrases [32] the *attB* sites cannot be predicted readily for Peaches and SkiPole, but BlastN analysis suggests that the *attB* sites for Eagle and Hope are the host tRNA-gly (Msmeg_4767) and tRNA-Arg (Msmeg_6349) genes respectively, which are the known integration sites for L5 [33] and BPs [8]. Similar analyses suggest that Island3 integrates into a tRNA-Thr (Msmeg_6152) gene. As noted above, we have been unable to identify a potential *attB* site for LeBron, and this is also true for Pumpkin and all of its Cluster E relatives (Cjw1, 244, Kostya and Porky).

CrimD and Angelica encode very similar tyrosine integrases (97.2% identical) whose closest relatives are those of the F1 subcluster phages (∼44% aa identity). BlastN analysis of the nucleotide sequence immediately upstream of these integrases shows that both phages contain a 23 bp sequence with identity to the 3′ half of the *M. smegmatis* tmRNA (Msmeg_2093), strongly suggesting that the tmRNA gene provides the *attB* site for these phages to integrate. While the use of tmRNA genes for phage integration has been noted previously [34] these are the first mycobacteriophages reported to use this type of site.

#### Evolution of genome anatomies in Cluster I phages

**Figure 5:**
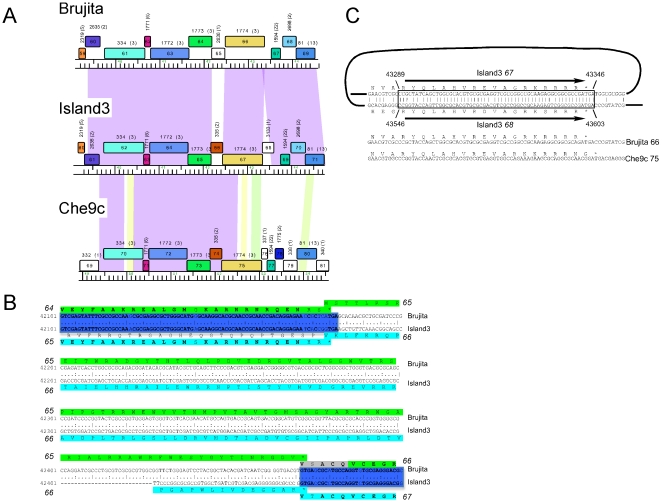
Evolution of Cluster I genome anatomies. **A.** Alignment of genome segments of phages Brujita, Island3, and Che9c. Genes are shown as colored boxes with gene names (a serial number based on that phage) inside the boxes and the pham number indicated above the box with the number of pham members in parentheses. Pairwise nucleotide sequence similarity is shown as colored areas between adjacent genomes, with strength of similarity according to the color spectrum, violet being the most similar, and red the least. **B**. Alignment of Brujita and Island3 shows that Brujita genes *64* and *66* are related to Island3 genes *65* and *67*, respectively, whereas Brujita gene *65* and Island3 gene *66* are distinctly different. The sequences in common are shown bold, and the common genes are shaded dark blue. **C.** Island3 genes *67* and *68* share a common 60 bp sequence at their 3′ ends. Brujita contains only a single copy of this sequence which represents a recombinant version that matches the upstream part of Island3 *67* and the downstream part of Island3 *68*. Che9c also shares the upstream sequence but is different downstream of gene *75* with sequence discontinuity close to the end of the gene.

Brujita, Island3, and the previously described Che9c constitute Cluster I, with the closely related Brujita and Island3 forming Subcluster I1. These two genomes are very closely related with only about 100 nucleotide differences across the syntenic parts of the genomes, which extend across the entire genome with two notable exceptions near the right ends (Fig. 5A, Fig. S1). Because the genomes are so closely related it is likely that the recombination events giving rise to these changes in genome anatomy occurred relatively recently in evolutionary time, warranting a closer examination to elucidate how they occurred.

The first of these differences is the substitution of Brujita gene *65* for Island3 gene *66* (Fig. 5A). The Island3 gene order is maintained in Che9c suggesting that this is the ancestral state (since independent acquisition of the same gene in Island3 and Che9c seems unlikely); Brujita gene *65* is an orpham and database searching reveals no close relatives. Alignment of the Island3 and Brujita genomes shows that the leftmost boundary between shared and unshared sequences occurs near, but not precisely at the termination codons of Brujita *64* and Island3 *65* (Fig. 5B), leading to amino acid changes in the C-terminal two residues. The stop codons appear in the same relative locations, but the sequences immediately to their right are unrelated at the nucleotide and amino acid sequence levels (Fig. 5B). The right boundary is close to the initiation codons of Brujita gene *66* and Island3 gene *67*. The genome substitution in Brujita can therefore be accounted for by two separate recombination events, one at each of the sequence discontinuities. Because we do not know the genome partner that contributed Brujita gene *65* we cannot rule out that recombination occurred between longer segments of homology by general recombination enzymes; however, the Brujita structure is consistent with illegitimate or non-sequence directed exchanges that occurred close to the gene boundaries, maintaining gene functions.

The second interruption in the synteny is the insertion of Island3 gene *68*; this gene has no counterpart in Brujita (Fig. 5A). The 85-residue predicted product (gp68) has no homologues, but the C-terminal 18 residues are closely related to the 18 C-terminal residues of the upstream gene *67* (Fig. 5C). This similarity is also reflected at the nucleotide sequence level (Fig. 5C), suggesting strongly that recombination occurred between ∼50 bp sequences to yield the observed structures. While this could have involved an intramolecular recombination event in an Island3-like parent to produce a Brujita deletion, it also could have occurred by intermolecular recombination between Brujita and an unknown partner genome to generate the Island3 structure. While we cannot readily distinguish between these possibilities, we note that both could be mediated by general recombination enzymes, a mechanism which is fundamentally similar to the process whereby short conserved boundary sequences are proposed to contribute to genomic mosaicism [21], [22]. This is the first example of such an event described in the mycobacteriophages.

#### Immune diversity in Cluster A phages

Expansion of the A cluster and the addition of new Subclusters A3 and A4 provides insights into the superinfection immunity systems among these phages. The basis of immunity has been established previously in L5 (Subcluster A2) and Bxb1 (Subcluster A1) [35], [36], [37]. Both encode a repressor protein (gp71 and gp69 respectively) containing a predicted helix-turn-helix (HTH) DNA binding domain that binds to 13 bp asymmetric DNA sites. There are multiple copies of this binding site located throughout the genomes (24 in L5 and 34 in Bxb1) – situated primarily in intergenic regions and oriented in one direction relative to transcription – and are referred to as stoperators [36], [37]. Their proposed role is to silence prophage transcription by blocking elongation of accidental expression events [36]. Bxb1 and L5 are heteroimmune, consistent with their having different consensus stoperator sequences (L5: 5′-GGTGGMTGTCAAG; Bxb1: 5′-GTTACGWDTCAAG, differences in Bxb1 underlined).

We have established the immunity patterns for the Cluster A phages that form stable lysogens (Table 6). Two of the phages – Bxz2 and KBG – form clear plaques from which we have been unable to recover stable lysogens, and the genome sequences of both appear to have single base substitutions in the repressor gene that would render them non-functional. The patterns of immunity closely reflect the subcluster designations of the phages derived from whole genome comparisons, such that phages within a subcluster are homoimmune, but each of the subcluster phages is heteroimmune with phages from other subclusters (Table 6). Currently Bxz2 is the sole member of subcluster B3 and although we are not able to recover lysogens, we did find that it is not subject to superinfection immunity by any other Cluster A phages (Table 6). We note that although it was previously reported that both L5 and D29 form plaques on a Che12 lysogen [38], they behaved as homoimmune in our experiments (Fig. S4).

**Table 6:** Immune specificities of Cluster A mycobacteriophages.

Lysogen	Bxb1	DD5	Jasper	*SkiPole*	Solon	Che12	L5	Pukovnik	*RedRock*	*Eagle*	*Peaches*
Beth	+	+	+	+	+	-	-	-	-	-	-
Bxb1	+	+	+	+	+	-	-	-	-	-	-
DD5	+	+	+	+	+	-	-	-	-	-	-
Jasper	+	+	+	+	+	-	-	-	-	-	-
KBG	+	+	+	+	+	-	-	-	-	-	-
Lockley	+	+	+	+	+	-	-	-	-	-	-
***SkiPole***	+	+	+	+	+	-	-	-	-	-	-
Solon	+	+	+	+	+	-	-	-	-	-	-
Che12	-	-	-	-	-	+	+	+	+	-	-
L5	-	-	-	-	-	+	+	+	+	-	-
Pukovnik	-	-	-	-	-	+	+	+	+	-	-
***RedRock***	-	-	-	-	-	+	+	+	+	-	-
D29	-	-	-	-	-	+	+	+	+	-	-
Bxz2	-	-	-	-	-	-	-	-	-	-	-
***Eagle***	-	-	-	-	-	-	-	-	-	+	+
***Peaches***	-	-	-	-	-	-	-	-	-	+	+
***LRRH***	-	-	-	-	-	-	-	-	-	-	-

Lysogens made from Cluster A phages are listed in the top row. Serial dilutions of phages (left column) were spotted onto lawns of lysogens and scored ‘+’ if the lysogen is immune to superinfection (i.e. homoimmune) and ‘-‘ if the lysogen is not immune (i.e. heteroimmunity). ‘Beth’ = Bethlehem, ‘LRRH’ = LRRHood. Lysogens were not successfully recovered from Bethlehem, KBG, Lockley, LRRHood, or Bxz2, and thus not listed in the top row. Bethlehem, Bxb1, DD5, Jasper, KBG, Lockley, SkiPole and Solon belong to Subcluster A1, Che12, L5, Pukovnik, Redrock and D29 belong to Subcluster A2, Bxz2 is the sole member of Subcluster A3, and Eagle and Peaches belong to Subcluster A4; LRRHood is in Subcluster C1. Newly isolated phages are indicated by bold italic type.

**Figure 6:**
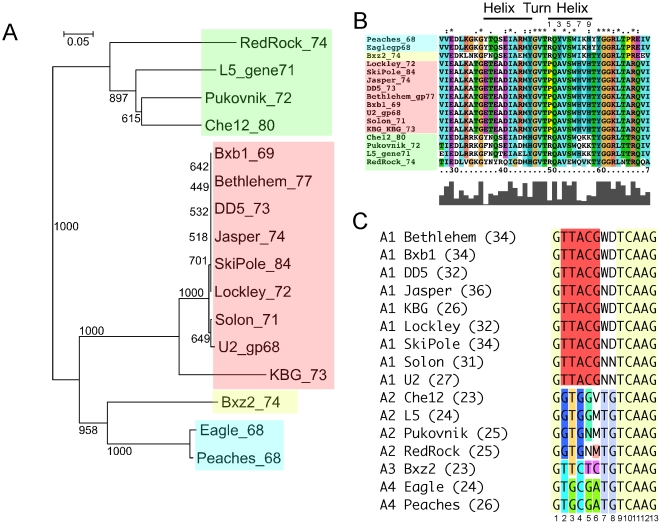
Determinants of immunity specificity in Cluster A genomes. **A**. Phylogenetic relationship of Cluster A repressors. The neighbor-joining (NJ) tree was drawn by NJPlot using output from an alignment in ClustalW; bootstrap values from 1000 iterations are shown. The repressor clades correspond closely to the subclustering of the genomes as indicated by color shading: Subcluster A1, red: A2, green, A3, yellow, A4, blue. **B**. The predicted helix-turn-helix motifs of the Cluster A repressor are aligned to show conserved and variant residues. The positions in the second recognition helix of the HTH motif are numbered. The Cluster assignation of the genome encoding the repressors is colored as in A. Bxz2 gp74 and KBG gp73 are included even though both contain frameshift mutations in the repressor gene. In KBG gp73 the mutation lies downstream of the HTH motif. There is a presumed single base deletion in Bxz2 at coordinate 44,987 upstream of the HTH motif, and we have used the ‘corrected’ sequence in the alignment that would results from inclusion of one additional bp at that position. **C.** Alignment of the consensus stoperator sites in Cluster A genomes. Consensus sequences were derived from alignments of putative operator and stoperator sites (shown in Fig. S4); mixed base consensus positions are indicated as W: A or T, D: A, G or T, V: C, G or T, M: A or C, N: A, C, G or T. Color shading indicates identical consensus positions.

Phylogenetic comparison of Cluster A repressors shows that they have generally evolved in concert with their overall genomes, with clades closely corresponding to cluster assignments (Fig. 6A). The A1 subcluster repressors are almost identical to each other, with the exception of KBG, primarily because of the frameshift mutation. The A2 subcluster repressors are somewhat more diverse, and the Eagle and Peaches (Subcluster A4) repressors are distinctly different from the other repressors, but very similar to each other (Fig. 6A).

The repressor-DNA recognition systems of the Cluster A genomes are non-canonical because of the asymmetry of the recognition sites [36], [37]. However, it seems likely that the second recognition helix of the repressor HTH motif contributes to specificity even though it may not be the sole determinant [39]. While recognition is not expected to follow a specific code for amino acid-base pair correspondence [39], comparison of variations among the Cluster A repressors and their sites may provide clues to immune specificity. For example, alignment of the putative helix-turn-helix motifs [35], [37] shows that the turn (GVT) is absolutely conserved in all of the repressors (Fig. 6B), as well as glutamine, valine, and tryptophan residues at positions 2, 4, and 6 respectively in the recognition helix (the second of the helix-turn-helix pair), and the YGG motif immediately following it (Fig. 6B). The main differences are thus at positions 1, 3, 5, and 7–10 in the recognition helix, which are thus implicated in DNA binding specificity as determinants of heteroimmunity.

We have examined each of the A cluster phages and identified 23–36 potential binding sites in each genome (Fig. S5). Although we do not have biochemical evidence to confirm that each site is bound by its cognate repressor, we note that all of the sites conform to a consensus sequence (Fig. 6C; Fig. S5) with no more than two nucleotide departures; they predominantly lie in intergenic regions or overlap gene ends, and in one orientation relative to the direction of transcription, as described for L5 and Bxb1 [36], [37]. Alignment of the 13-base asymmetric consensus sequences reveals those positions that are invariant and those that vary and are thus good candidates for discrimination of repressor binding and determination of specificity (Fig. 6C). The invariant positions are 1, 9, 10, 11, 12 and 13 (Fig. 6C), and we note for example that there is not a single departure from a G in any of the 453 sites identified here in position 1, there are only two departures from the A at position 12, five sites have a base other than a G in position 13, and nine sites have a base other than a T at position 9. The least conserved of the consensus nucleotides is the C at position 10 (58 sites have a different base pair) (Fig. S5). Because positions 7 and 8 show considerable variability in the A1 phages (note the use of W, D, and N alternatives), it seems unlikely that they make major contributions to binding of that repressor; 5′-TG is well conserved in the A2, A3, and A4 subcluster genomes, but TG is also the most common dinucleotide in all of the A1 genomes (Fig. 6C, Fig. S5). We therefore predict that positions 2–6 encompass most of the site specificity determinants.

A comparison of the site sequences and the repressor helix-turn-helix motif reveals some interesting correlations, including some very apparent ones involving positions 2, 3, and 4 in the stoperator nucleotide sequences. For example, all of the sites contain a T at position 2 with the exception of the Cluster A2 phages, which have a G in that position (Fig. 6C). In the putative recognition helix of the repressor HTH motif there is only one position that mirrors this distribution, which is residue 9, a histidine in all members except for Cluster A2, and a lysine residue in members of Subcluster A2 (Fig. 6B). This suggests the possibility that residue 9 of HTH may be specifically involved in discriminating between stoperator sites with nucleotide differences at position 2; we note that there is not a single departure from the respective consensus sequence in any of the constituent sites at this position (Fig. S5). It has also been shown that substitution of a T at position 2 in the L5 site strongly inhibits repressor binding [40]. A similar case can be made for position 3 in the binding site and residue 10 in the recognition helix, where Subcluster A4 sites have a G and all others have a T; in the repressor, residue 10 is a threonine in all repressors except for Cluster A4, in which it is a tyrosine. We also note that at site position 4, which is an A in the A1 sites, and either a C or a G in all other sites, multiple residues in the HTH may be involved in binding specificity; as residue 1 of the recognition helix in the HTH is a proline in all of the A1 repressors, but an arginine in all of the rest; while residue 3 of the HTH is a tyrosine residue in Subclusters A3 and A4 but an alanine in Subclusters A1 and A2, correlating with a C base at position 4 in A3 and A4 sites, and either a G or an A in Subclusters A1 and A2. Substituting a C at position 4 in the L5 site prevents repressor binding [40]. The other variable positions in the HTH are at positions 5, 7, and 8, and while these may contribute to specificity, there are no clear correlations with site differences. Experimental dissection is clearly required to test the contributions of any of these correlations to immune specificity.

#### Unexpected presence of a Cluster A1 phage repressor in Cluster C phage LRRHood

**Figure 7:**
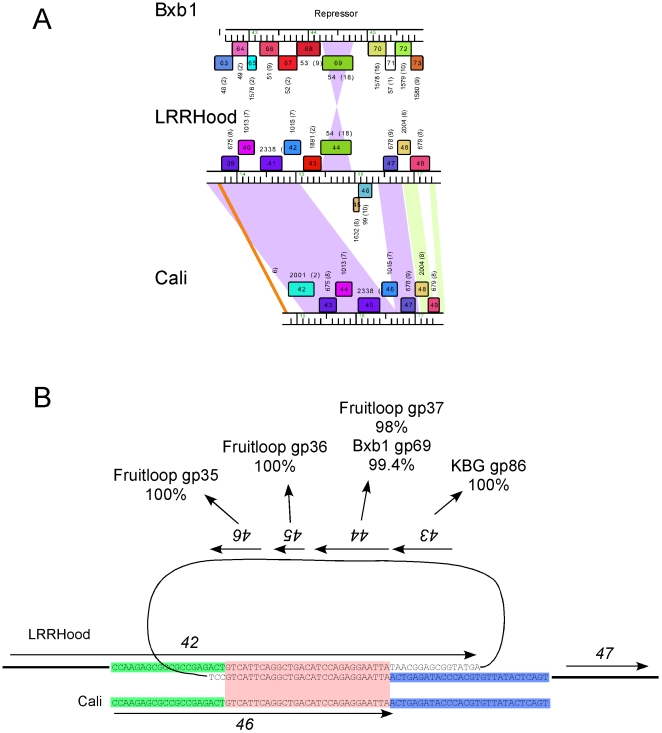
Immunity theft in mycobacteriophage LRRHood? **A.** LRRHood is a Cluster C1 phage that contains a ∼1.4 kbp insertion relative to other C1 phages such as Cali. The insertion contains gene *44* encoding a repressor with >99% identity to Subcluster A1 repressors such as Bxb1 gp69. LRRHood does not contain any copies of the repressor binding site, and we propose that its gene *44* functions to protect LRRHood-infected cells from superinfection by Subcluster A1 phages. **B**. LRRHood gene *44* is one of four genes (*43*–*46*) that are absent from the related phage Cali. The 1.35 kbp additional DNA in LRRHood is flanked by 29 bp direct repeats, of which there is only a single copy in Cali and other Cluster C1 phages. Presumably, either LRRHood acquired this 1.35 kbp segment from another phage by recombination within the 29 bp region, or it has been lost from each of the other Cluster C1 phages. The 29 bp repeat is shown in the red box and is present once in Cali at the extreme 3′ end of gene *46*, and twice in LRRHood in the end of gene *42* and upstream of gene *47*. The upstream and downstream regions common to LRRHood and Cali are shown in green and blue respectively.

Two of the newly sequenced phages, ET08 and LRRHood are clearly members of Subcluster C1. Although LRRHood shares close similarity to other Subcluster C1 phages, it contains a 1.35 kbp segment that is absent from closely related genomes such as Cali (Fig. 7A). This segment includes four putative genes, *43*–*46*, all of which have homologues located in other mycobacteriophage genomes. The homologues of genes *45* and *46* are in Cluster F1 genomes, and gp43 has a single homologue that is present in the Cluster A1 phage KBG (gp86). Interestingly, LRRHood gp44 is a close relative to the repressor proteins of Cluster A phages discussed above, having only a single amino acid difference with Bxb1 gp69 (an Arg for His substitution at position 161, away from the HTH motif); there is strong experimental support for the repressor function of Bxb1 gp69 [37]. The DNA sequence of LRRHood gp44 is 99% identical to Bxb1 gene *69* suggesting that it has been acquired recently in evolutionary time. However, none of the Cluster C phages have previously been shown to be temperate, and we have not been successful in isolating stable LRRHood lysogens; we note also that no Cluster C genomes encode either an integrase or other functions associated with prophage maintenance. The presence of a putative repressor gene of the Cluster A type is therefore highly unexpected.

Further examination reveals that the 1.35 kbp region of LRRHood containing genes *43*–*46* is flanked by 29 bp direct repeats (Fig. 7B). There is only a single copy of the sequence in other Subcluster C1 phages, suggesting either that all of the Subcluster C1 phages have lost a 1.35 kbp segment *via* recombination between the two repeat copies, or alternatively that LRRHood acquired this DNA segment by recombination with a second phage partner of unknown origin (Fig. 7B). We favor the second explanation although this 29 bp sequence is absent from all phages other than the Subcluster C1 and thus it is not possible at this time to reconstruct how this presumed acquisition occurred.

Because Cluster A genome repressors are associated with multiple operator and stoperator binding sites, we examined LRRHood for the presence of these. However, we were not able to identify a single copy of a 13 bp sequence corresponding to the consensus sequence for stoperators of the Cluster A1 type (Fig. 6) and it therefore seems unlikely that LRRHood has acquired gene *44* as a core component to its own immunity system. We also showed that LRRHood is not subject to immunity from any Cluster A phage (Table 6). We therefore propose that LRRHood has acquired gp44 and retained it in its genome because it confers protection to LRRHood-infected cells against superinfection by Cluster A1-like phages, presumably during lytic growth. This is the first example of apparent repressor theft that we are aware of in any characterized bacteriophage.

We note that the data do not allow us to rule out an interesting alternative interpretation of gene *44*′s presence in the LRRHood genome. That is, it is possible that gene *44* may not be providing any selective benefit to LRRHood and is found there simply because it, together with the three associated genes (*43*, *45* and *46*), entered the genome adventitiously very recently and has not yet been removed by random deletion.

#### Identification of new intein insertions in phage ET08

Several mycobacteriophage inteins, sequences able to splice out of a protein precursor, have been described previously, one of which has been shown to have novel biochemical activities [41]. Comparison with other Cluster C genomes and searching against the InBase intein database (http://www.neb.com/neb/inteins.html) showed that ET08 has at least five inteins within gene products gp3, gp79, gp202, gp239 and gp248, the most of any mycobacteriophage sequenced to date. ET08 gp3 and its homologues are predicted to encode nucleotidyltransferases, and the exteins are very closely related (92–96% amino acid identity). Two other Cluster C orthologs (Cali gp3 and LRRHood gp3) contain a related intein, but it shares only 67% amino acid identity, suggesting that ET08 gp3 has a recent intein insertion. Two of the ET08 intein-containing proteins (gp202 and gp239) are nearly identical across their spans to homologues in other Subcluster C1 phages, although some of the related proteins are intein-less. ET08 gp202 shares 99% amino acid identity with intein-containing homologues in ScottMcG gp208, Spud gp208, Catera gp206 and Rizal gp206, and the exteins are 98–99% identical to the intein-less homologues Bxz1 gp204, LRRHood gp207, and Cali gp207; more distantly related versions of this intein are present in the unrelated genomes Omega gp11 and Kostya gp19 (49% and 39% identity respectively). ET08 gp239 shares 98% identity with Spud gp245, Rizal gp242, Cali gp243, LRRHood gp245, ScottMcG gp245 and Catera gp242, and the exteins are 99–100% identical to Bxz1 gp239.

**Figure 8:**
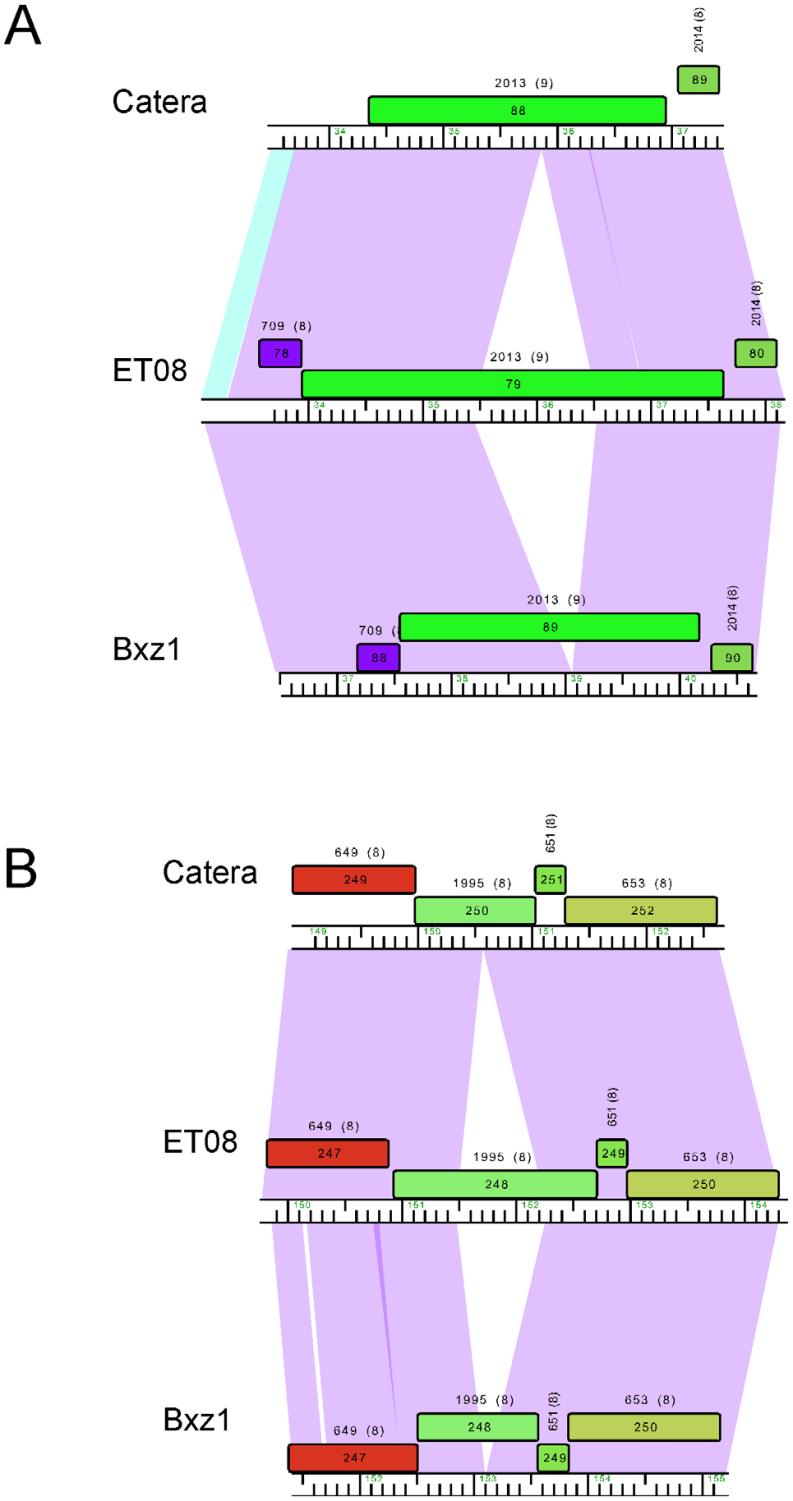
Novel intein insertions in mycobacteriophage ET08. ET08 is a Subcluster C1 phage and encodes two products, gp79 (**A**) and gp248 (**B**) that contain intein insertions that are absent from other C1 genomes such as Catera and Bxz1.

Two of the ET08 inteins (in gp79 and gp248) appear to have been acquired recently and are absent from all other homologues in other phages (Fig. 8). The extein homologues are >95% identical to their intein-less Subcluster C1 homologues, and there are no close relatives of either intein in any of the sequenced mycobacteriophage genomes. There is an intein distantly related to the ET08 gp79 intein in Corynebacterium phage P1201 (25% amino acid identical), and the ET08 gp248 intein is a distant relative (∼25% identity) of the inteins present in ET08 gp239 and ET08 gp79 (23% identity). With the exception of ET08 gp3, there are no known or predicted functions of the ET08 intein-containing proteins, although because inteins are commonly associated with essential gene functions [42], we predict that all of these genes are required for ET08 propagation.

#### A new MPME1 insertion in phage Hope

Mycobacteriophage Hope is a member of Cluster G and all of the Cluster G phages, BPs, Halo, Angel, and Hope are extremely similar at the nucleotide sequence level [8]. Prior analysis of Angel, Halo and BPs revealed the presence of a family of novel ultra-small mobile mycobacteriophage elements (MPMEs) of which there are two related types, MPME1 and MPME2 [8]. Separate insertion sites were identified in BPs and Hope, although Angel is devoid of these elements.

**Figure 9:**
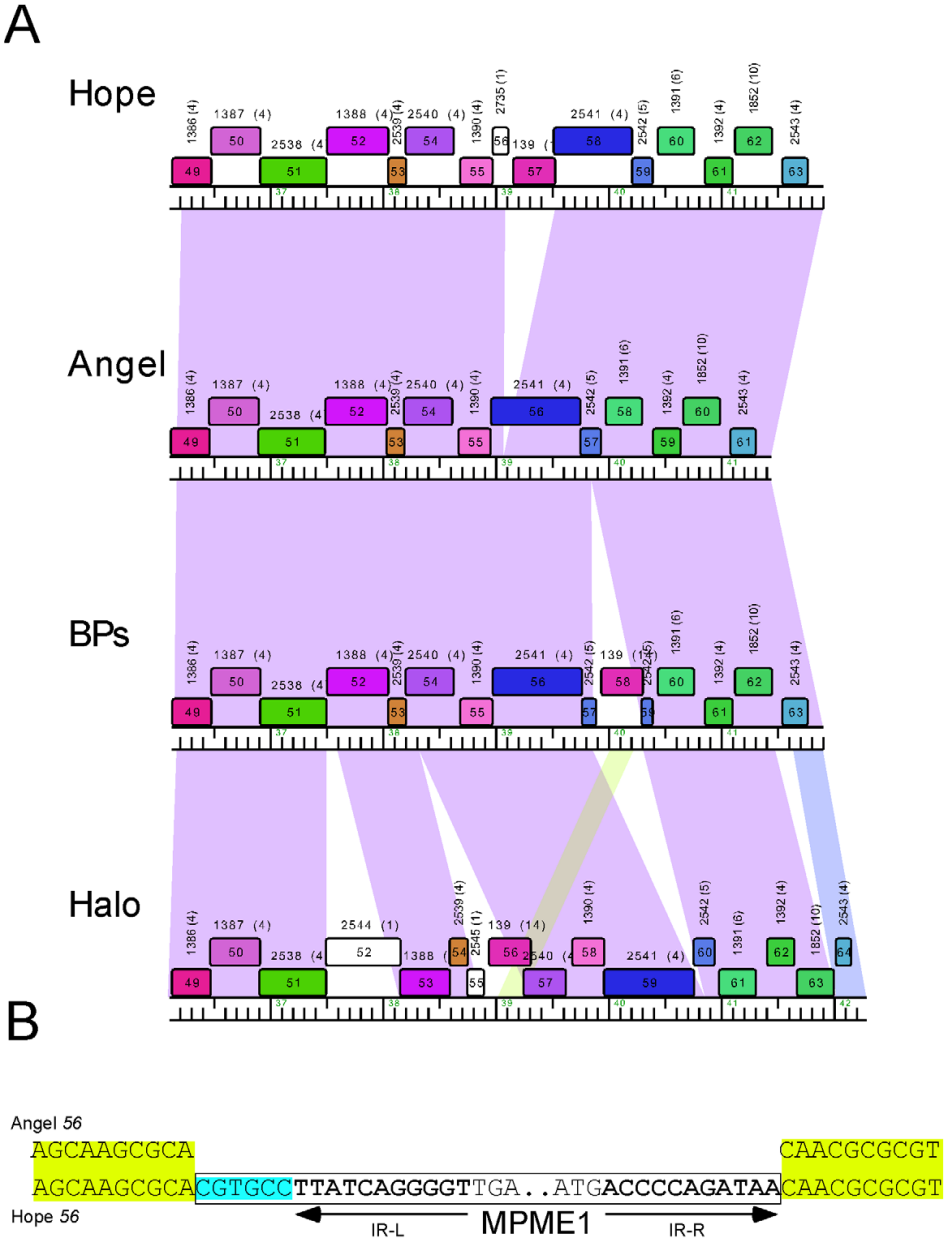
MPME1 insertion in mycobacteriophage Hope. **A.** Alignment of the four closely related phages in Cluster G reveals insertions of MPME elements in BPs, Hope, and Halo. Hope contains an MPME1 insertion at a new site corresponding to Angel *56*. **B**. Comparison of the Hope and Angel sequences reveals the pre-integration site; left and right inverted repeats (IR-L and IR-R) are shown in bold type. At the left end there is the presence of an atypical 6 bp insertion (shown in turquoise) between IR-L of MPME1 and the target.

Comparison of Cluster G genome maps shows that Hope contains an MPME1 insertion, although it is inserted at a novel genomic site within the related Angel gene *56*, near the 5′ end (Fig. 9A). The much larger 3′ end of Angel gene *56*, encoding residues 38–263 of gp56, is conserved in Hope as gp58. The Hope MPME1 is 439 bp in length and identical in sequence to the MPME1 element in BPs. The identity of the Angel and Hope sequences in this region enables identification of the precise site of insertion, and we note that like all other MPME insertion events, there is addition of a 6 bp sequence between IR-L of the MPME1 and the target sequence (Fig. 9B). This 6 bp sequence is different from any of those found at other MPME insertion events, and its origin remains a mystery. Although the functions of Angel gp56 and its full-length homologues in Halo and BPs are not known, the MPME1 insertion in Hope suggests that the N-terminal domain of Angel gp56 is not essential for protein function. It is notable that three of the four closely related Cluster G genomes contain independent MPME insertions, consistent with the interpretation that these elements are actively mobile and move at reasonable frequencies.

#### Gene swapping in Subcluster B1 genomes

**Figure 10:**
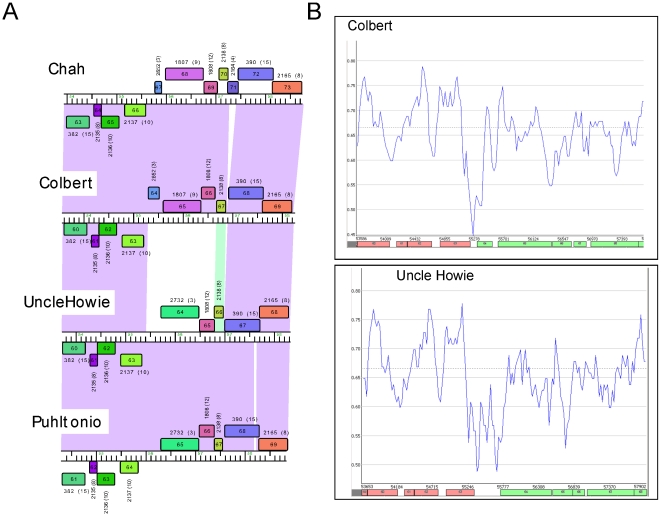
Gene swapping in Cluster B1 genomes. **A.** Alignment of the Cluster B1 genomes shows a swap of genes between Chah genes *63* and *73* and their homologues. Segments of genome maps of phages Chah, Colbert, UncleHowie and Puhltonio are shown with regions of nucleotide similarity identified by colored shading between them; shading reflects degrees of similarity with the color spectrum, such that violet is most similar and red the least similar. **B.** A reduction in GC% content in an intergenic region between Colbert genes 63 and 64 and UncleHowie genes *63* and *64* suggests the presence of regulatory elements notwithstanding the swapping of the downstream genes.

Five of the newly sequenced genomes, UncleHowie, Puhltonio, Fang, Scoot17C, and Colbert are members of Subcluster B1, more than doubling the size of this subcluster, joining the three B1 genomes reported previously. Subcluster B1 is notable in that its genomes show a high degree of similarity to each other (pairwise ANI values>98%) [7], and this remains true after the addition of the three new genomes. However, alignment of the B1 genome maps reveals notable differences between these genomes. The most apparent difference is a region of ∼2 kbp in which two alternative segments are observed; one in UncleHowie, Puhltonio, and Fang, and a second in PG1, Orion, Scoot17C, Chah, and Colbert (Fig. 10). The first group contains two ORFs of unknown function (UncleHowie genes *64* and *65*) as well as an upstream ∼380 bp region that is non-coding, AT-rich, and likely contains control sequences. Fang contains an additional gene, *67*, which is also of unknown function. The second group contains two genes of similar size to UncleHowie *64* and *65* (genes *65* and *66* in Colbert), as well as an upstream gene, *64*; all are of unknown function. Interestingly, the sequence upstream of Colbert *64* also contains an AT-rich region that is consistent with a regulatory function (Fig. 10B). These mosaic substitutions with the inclusion of predicted regulatory sequences are reminiscent of previously described phage morons, DNA elements inserted between a pair of gene that are contiguous in other phages [17]. The functional consequences of this genetic swap are not known.

#### HNH Homing Endonuclease Elements in Cluster E genomes

**Figure 11:**
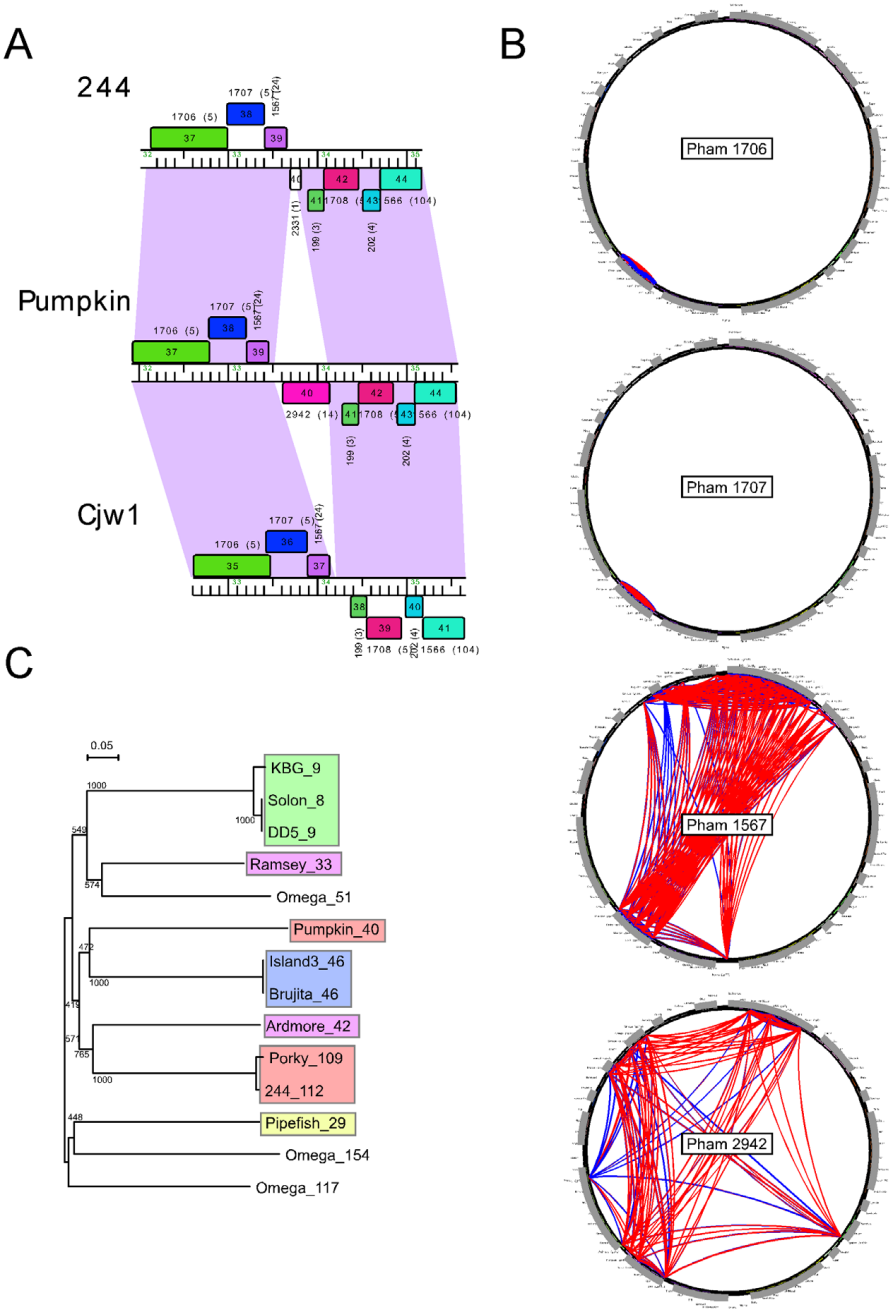
Acquisition of an HNH homing endonuclease in mycobacteriophage Pumpkin. **A.** Alignment of the Cluster E genomes 244, Pumpkin, and Cjw1 reveals the presence of a Pumpkin gene (*40*) that is absent from other Cluster E genomes. **B.** Phamily circles of Phams 1706, 1707, 1567, and 2942, which include Pumpkin genes 37, 38, 39, and 40. Pham1706 and 1707 have members exclusively within Cluster E genomes. Pham 1567 is more widely distributed with members in Subcluster A1, some in A2, F2, and singleton genomes, and is functionally a glutaredoxin. Pham 2942 is a homing endonuclease and is broadly distributed among the mycobacteriophages. Each of the 80 genomes is shown on the circumference of each circle – arranged by cluster – with arcs indicating pairs of genomes containing a pham member; thicker arcs indicate closer similarity. Red and blue arcs show BlastP and ClustalW comparisons respectively. **C.** Phylogenetic reconstruction of Pham2942. All members are distantly related, and Pumpkin gp40 probably was acquired independently from other Cluster E acquisitions (Porky gp109, 244 gp112). Members are colored according to cluster of parent genome: A1, green; F1, purple; E, orange; I1, blue; B3, yellow; Omega is a singleton. The neighbor-joining (NJ) tree was drawn by NJPlot using output from an alignment in ClustalW; bootstrap values from 1000 iterations are shown.

One of the newly sequenced genomes, Pumpkin, is a member of Cluster E, a group of five genomes with substantial similarity but with more syntenic interruptions than seen in the Subcluster B1 genomes discussed above. Some of these interruptions are caused by genes whose products are strongly predicted to contain HNH homing endonuclease domains, and are thus expected to be mobile within phage populations and contribute to genome mosaicism. An example is presented by Pumpkin gene *40*, which is absent from other Cluster E genomes at that syntenic location (Fig. 11A). The genome mosaicism is illustrated by the phamily circles of Phams 1706, 1707, 1567 and 2942, to which the four consecutive Pumpkin genes *37*, *38*, *39*, and *40* belong (Fig. 11B). Phams 1706 and 1707 are restricted to pham members within the Cluster E genomes, whereas Phams 1567 and 2942 are much more widely distributed. Pham 1567 encodes a putative glutaredoxin protein and is presumably widely distributed because it confers an advantageous function. In contrast, Pham 2942 presumably enjoys a wide distribution because of its ability to self-mobilize. We note that two other Cluster E genomes, 244 and Porky, also encode Pham 2942 members, although they are distantly related to Pumpkin gp40 (Fig. 11C) and are located at a remote location in the right arms of the genomes. The high sequence diversity of this pham (∼30% amino acid identity) suggests that Pumpkin did not acquire gp40 from other Cluster E genomes, or any other close relative of the currently sequenced genomes.

### 3. Summary

Expansion of the current collection of sequenced mycobacteriophage genomes clearly demonstrates the remarkable genomic diversity of a set of bacteriophages known to infect a common host, *Mycobacterium smegmatis*. The addition of these 18 genomes allowed us to examine the mechanism and evolution of the Cluster A repressor/stoperator system and analyze the distribution of mobile elements, inteins, and HNH endonucleases in mycobacteriophages. Even with 80 sequenced genomes, it appears that we have still sampled only a proportion of the total mycobacteriophage population in the environment, and it remains difficult to predict what pattern of new genomes might be expected; novel genomes clearly remain to be discovered. The expansion of the geographical locations from which the phages were isolated does not provide any strong evidence for geographical restriction of phage subtypes, as most of the newly sequenced genomes are identifiably related to those recovered predominantly from the Western Pennsylvania area. Likewise, an examination of the temporal distribution of phage type isolation does not yield any obvious pattern at this time. In both cases, it is likely that we have drastically under-sampled the pool of possible genomes, and further isolates will need to be analyzed in order to draw any conclusions as to the roles space and time play in phage distribution.

Finally, twelve of the new phages isolated, described, and analyzed here resulted from a new program implemented by the HHMI Science Education Alliance – as a component of the National Genomics Research Initiative (NGRI) – in which freshman undergraduate students at 12 colleges and universities engaged in phage discovery and genomics. The other six phages were isolated within the Phage Hunters Integrating Research and Education (PHIRE) program at the University of Pittsburgh [16], [43], [44] that provided the framework for the SEA initiative. The successful isolation, sequencing, and comparative genomic analysis – together with the considerable insights into viral diversity and into the evolutionary mechanisms that shape genome architectures reported here – attests to the effectiveness of phage discovery and genomics for introducing freshman undergraduates to authentic scientific research in a broad range of institutional contexts.

## Materials and Methods

### Phage isolation and purification

All phages isolated at the Pittsburgh Bacteriophage Institute (PBI) were isolated by co-plating of soil extracts, prepared with Phage Buffer (10 mM Tris/HCl pH 7.5, 10 mM MgSO_4_, 1 mM CaCl_2_, 68.5 mM NaCl), and *M. smegmatis* mc^2^155. The soil extract was filtered through a 0.22 µm filter and 50 µl of this sample was plated with 0.5 ml late-exponential-phase *M. smegmatis* mc^2^155 in 4.5 ml 0.35% mycobacterial top agar (MBTA) with 1 mM CaCl_2_. Phages isolated through the SEA Alliance were either found through direct plating or by enrichment cultures. Phages isolated by direct plating were found using the same protocol as PBI. Phages found via enrichment were isolated as follows: approximately one gram of a soil sample and 5 ml of late log/early stationary phage *M. smegmatis* mc^2^155 was incubated in 50 ml of 7H9 plus 1 mM CaCl_2_ at 37°C shaking for 24 hours. Any remaining cells were then pelleted by centrifugation, and the supernatant was filter-sterilized through a 0.22 µ filter. The sterilized filtrate was serially diluted with phage buffer and plated as above with MBTA and *M. smegmatis* mc^2^155. For both direct plating and enrichment plates, the MBTA/phage/bacterial mixture was distributed evenly on a plate of 7H10 agar (Difco) supplemented with carbenicillin, cycloheximide, 1 mM CaCl_2_ and 10% albumin dextrose complex (ADC). Plaques were picked into phage buffer and replated with bacteria for several rounds of infection to purify phage isolates. After several rounds of purification, high-titer phage stocks were prepared at PBI using CsCl gradients and ultracentrifugation (Sarkis & Hatfull, 1998). DNA from SEA Alliance phages was harvested by treating 10 ml of a filtered phage crude lysate (about 10^9^ plaque forming units (pfu)/ml) with RNaseA and DNaseI for 30 min at 37°C followed by a 60-minute incubation at room temperature. Intact particles were then precipitated with 30% polyethylene glycol (PEG) 8000/3.3 M NaCl overnight at 4°C and then pelleted by centrifugation at 10000 g for 20 min. DNA was extracted from the phage pellet using the Promega Wizard DNA clean-up kit as per manufacturer's instructions.

### Phage genome sequencing

At PBI, double-stranded DNA was phenol extracted from the dialyzed CsCl banded phages, and pyro-sequenced at the University of Pittsburgh Genomics and Proteomics Core Laboratories (UPGPCL) as described previously [8]. SEA-associated phages were sequenced by the Joint Genome Institute using Sanger Sequencing on a 3730 ABI capillary sequencer. Finishing work for both PBI and SEA phages was done at PBI using Sanger sequencing on a 3730 ABI capillary sequencer. Sequence data were assembled using NewBler assembler and Consed. Finished sequences were analyzed and annotated in genome editors including DNAMaster (http://cobamide2.bio.pitt.edu), G Browse [45], Apollo [46], and the University California Santa Cruz Genome Browser [47]; Glimmer [48], GeneMark [49], tRNA ScanSE [50], Aragorn [51], and Programmed Frameshift Finder [52] were used to identify genome features. Genes were assigned to phams, and genome maps and phamily circle diagrams were drawn using Phamerator [7], [16]. The threshold parameters of 32.5% identity with ClustalW and a BlastP E-value of 10^−50^, are different to those used previously, and were derived by optimizing pham assembly over a range of possible values (S.G.C., R.W.H., G.F.H., manuscript in preparation). GenBank accession numbers are shown in Table 1. DotPlots were made using Gepard [29].

### Electron Microscopy

At the University of Pittsburgh, electron microscopy was performed by placing a suspension of virion particles on a grid with a carbon-coated nitrocellulose film, staining with 2% uranyl acetate, and examining the grid in an FEI Morgagni 268 transmission electron microscope equipped with an AMT digital camera system. Schools participating in the SEA program used locally available electron microscopy resources to obtain micrographs.

## Supporting Information

Figure S1 **Genome maps of 18 newly sequenced mycobacteriophage genomes.** Genome maps for each of the 18 newly sequenced mycobacteriophage genomes were generated using Phamerator. The order of the genomes from top to bottom is according to cluster, numerically[#alphabetically?] from A to singleton. Genes are represented as colored boxes above (rightwards transcribed) or below (leftwards transcribed) each genome, and are colored according to their pham assignation. The pham number is shown above or below each gene with the number of pham members in parentheses. The nucleotide similarity between adjacently displayed genomes is represented by the coloring between genomes, with the strength of the relationship represented according to the color spectrum, with violet being the most similar.
(PDF)

Figure S2 **Nucleotide sequence comparison of 17 Cluster A mycobacteriophage genomes.** Nucleotide sequences of all 17 Cluster A genomes were concatenated into a single file and compared against themselves using Gepard [29]. The 17 genomes can be grouped into four subclusters, A1–A4, as shown below the dotplot. Subcluster assignments are warranted as follows. SkiPole is assigned to subcluster A1 and shares a minimum of 87.5% average nucleotide identity (ANI) with other A1 phages; RedRock is assigned to subcluster A2 and shares a minimum of 75.1% ANI with other A2 phages (although we note that the diversity of this subcluster is substantially greater than within A1); phages Eagle and Peaches are very similar to each other (97.5% ANI) but have no more than 70.4% ANI with any other Cluster A phage and we assigned them to a new A4 subcluster; phage Bxz2 was previously assigned to Subcluster A2, but it shares no more than 70.4% ANI with any other genome, and is therefore re-assigned as the founding member of the new subcluster A3.
(PDF)

Figure S3 **Annual changes in types of isolated mycobacteriophages. A.** Annual changes in the total accumulated numbers of sequenced mycobacteriophages are shown by cluster. Date reflects date of isolation. The accumulated numbers of subclusters within Cluster A (**B**) and Cluster B (**C**) are also shown. Some weak patterns in phage isolation are evident. For example, by 2004 there were seven Subcluster C1 phage genomes, but then no more were isolated until 2008. Likewise, by 2006 there were more A1 subcluster genomes than any other, but no new ones have been isolated since then. In contrast, the number of Subcluster B1 phages more than doubled during this period. Because of the high genetic diversity of the mycobacteriophage population, the sizes of each of the cluster or subcluster groups is small and statistically significant numbers of phages will not be available until there is a 5 to10 –fold increase in the total number of sequenced mycobacteriophage genomes. Subtle changes in isolation methods – including enrichment versus direct plating – could influence the types of phages recovered, as well as changes at the population level.
(PDF)

Figure S4 **Immunity patterns of Bxb1, DD5, Jasper, SkiPole, Che12 and L5**. Ten-fold serial dilutions of phages were spotted onto either a non-lysogen or a lysogen as indicated, and incubated. Che12 and L5 are homoimmune, and Bxb1, DD5, Jasper, and SkiPole are homoimmune.
(PDF)

Figure S5 **Derivation of consensus sequences for Cluster A genome stoperator sites.** The 13 bp consensus stoperator sequence for each of 16 Cluster A phages was derived by searching for related sequences in each genome. The base distribution of each of the 13 positions is shown for each genome, with the consensus shown below. A single consensus base was assigned if it occurred in greater than 85% of the sites, and if not then standard abbreviations are used, W: A or T; D: G, T or A; V: G, C or A; M: A or C; N: anything. All sites listed have no more than two deviations from the consensus. For L5, the sites are the same as those described previously [36] where there is experimental evidence supporting repressor binding. For Bxb1, the sites listed are the same as described previously [37] except for inclusion of one additional potential site identified at coordinates 508–520 on the complement strand, and omission of one site previously identified at coordinates 44,803–44,815 that has more than two departures from the consensus. The previously described site at 48,867–48,855 has more than two departures from the consensus but has been show experimentally to be bound by the Bxb1 repressor [37]. We note that in the absence of experimental evidence to address the binding specificity of each of the A1 repressors that these compilations are likely to have omitted some additional binding sites while including some that may be recognized poorly by the cognate repressor. Phage D29 was omitted from the list since it does not express an active repressor, although a consensus stoperator sequence has been described previously which is identical to that of L5 [53].
(XLSX)

Table S1 **Author contributions.**
(XLSX)

Table S2 **Pham assignment table.**
(XLS)
