# Supplementary figures and images for: Expanding the Diversity of Mycobacteriophages: Insights into Genome Architecture and Evolution
Source: PLoS One. 2011 Jan 27;6(1):e16329. doi: 10.1371/journal.pone.0016329 (PMC3029335; doi:10.1371/journal.pone.0016329)

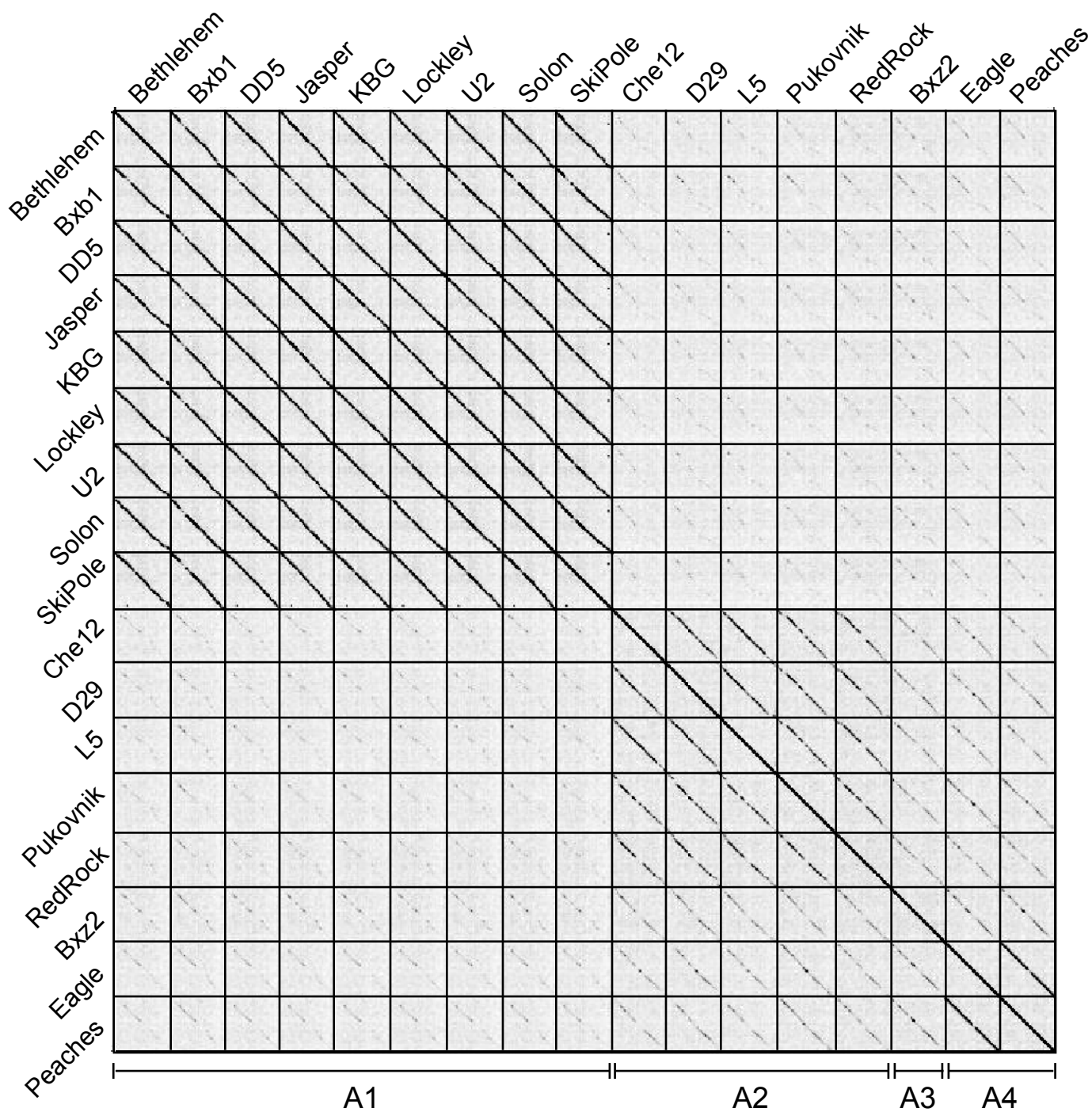

Figure S2

Supplement: Figure S2 — Nucleotide sequence comparison of 17 Cluster A mycobacteriophage genomes. Nucleotide sequences of all 17 Cluster A genomes were concatenated into a single file and compared against themselves using Gepard [29]. The 17 genomes can be grouped into four subclusters, A1–A4, as shown below the dotplot. Subcluster assignments are warranted as follows. SkiPole is assigned to subcluster A1 and shares a minimum of 87.5% average nucleotide identity (ANI) with other A1 phages; RedRock is assigned to subcluster A2 and shares a minimum of 75.1% ANI with other A2 phages (although we note that the diversity of this subcluster is substantially greater than within A1); phages Eagle and Peaches are very similar to each other (97.5% ANI) but have no more than 70.4% ANI with any other Cluster A phage and we assigned them to a new A4 subcluster; phage Bxz2 was previously assigned to Subcluster A2, but it shares no more than 70.4% ANI with any other genome, and is therefore re-assigned as the founding member of the new subcluster A3. (PDF) [file pone.0016329.s002.pdf]

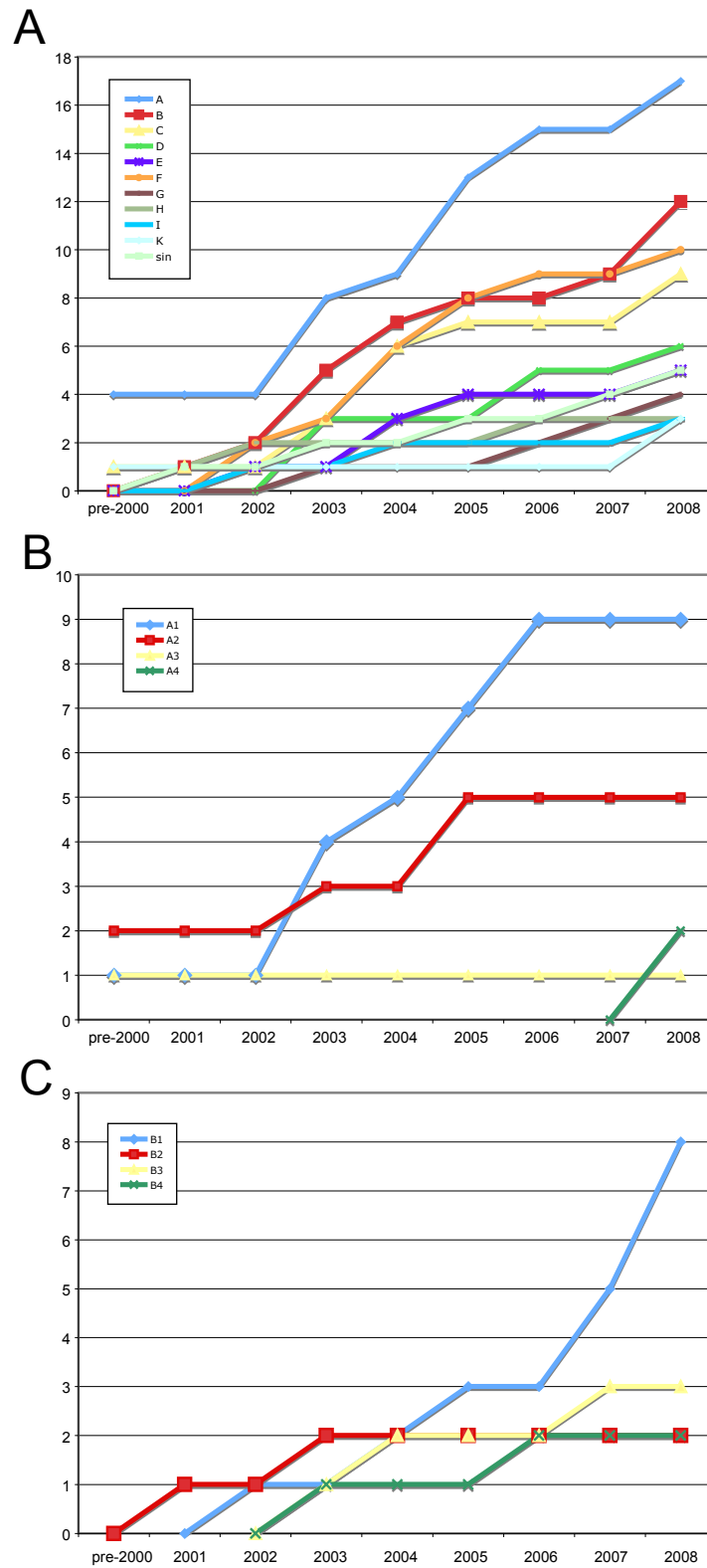

Figure S3

Supplement: Figure S3 — Annual changes in types of isolated mycobacteriophages. A. Annual changes in the total accumulated numbers of sequenced mycobacteriophages are shown by cluster. Date reflects date of isolation. The accumulated numbers of subclusters within Cluster A (B) and Cluster B (C) are also shown. Some weak patterns in phage isolation are evident. For example, by 2004 there were seven Subcluster C1 phage genomes, but then no more were isolated until 2008. Likewise, by 2006 there were more A1 subcluster genomes than any other, but no new ones have been isolated since then. In contrast, the number of Subcluster B1 phages more than doubled during this period. Because of the high genetic diversity of the mycobacteriophage population, the sizes of each of the cluster or subcluster groups is small and statistically significant numbers of phages will not be available until there is a 5 to10 –fold increase in the total number of sequenced mycobacteriophage genomes. Subtle changes in isolation methods – including enrichment versus direct plating – could influence the types of phages recovered, as well as changes at the population level. (PDF) [file pone.0016329.s003.pdf]

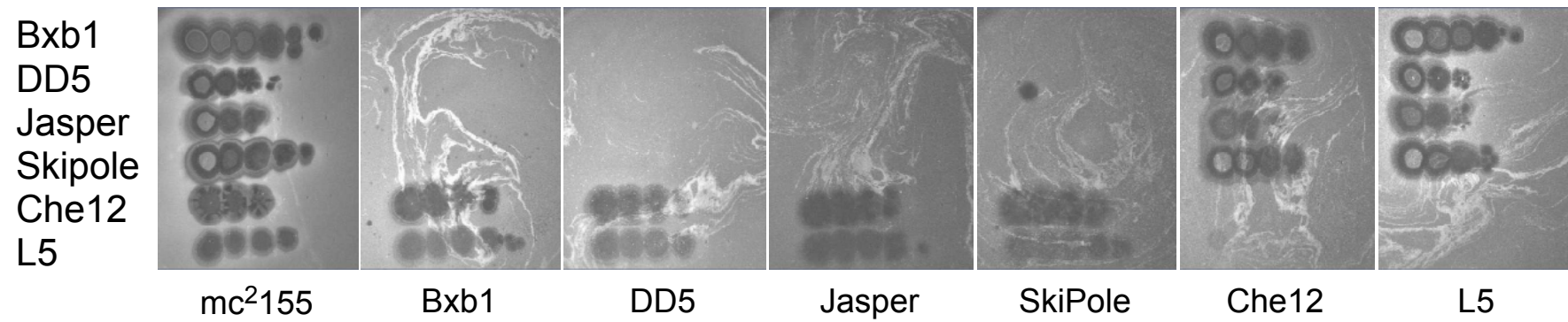

Figure S4

Supplement: Figure S4 — Immunity patterns of Bxb1, DD5, Jasper, SkiPole, Che12 and L5. Ten-fold serial dilutions of phages were spotted onto either a non-lysogen or a lysogen as indicated, and incubated. Che12 and L5 are homoimmune, and Bxb1, DD5, Jasper, and SkiPole are homoimmune. (PDF) [file pone.0016329.s004.pdf]
