# Supplementary material for: Expanding the Diversity of Mycobacteriophages: Insights into Genome Architecture and Evolution
Source: PLoS One. 2011 Jan 27;6(1):e16329. doi: 10.1371/journal.pone.0016329 (PMC3029335; doi:10.1371/journal.pone.0016329)
Supplement: Figure S1 — Genome maps of 18 newly sequenced mycobacteriophage genomes. Genome maps for each of the 18 newly sequenced mycobacteriophage genomes were generated using Phamerator. The order of the genomes from top to bottom is according to cluster, numerically[#alphabetically?] from A to singleton. Genes are represented as colored boxes above (rightwards transcribed) or below (leftwards transcribed) each genome, and are colored according to their pham assignation. The pham number is shown above or below each gene with the number of pham members in parentheses. The nucleotide similarity between adjacently displayed genomes is represented by the coloring between genomes, with the strength of the relationship represented according to the color spectrum, with violet being the most similar. (PDF) [file pone.0016329.s001.pdf]

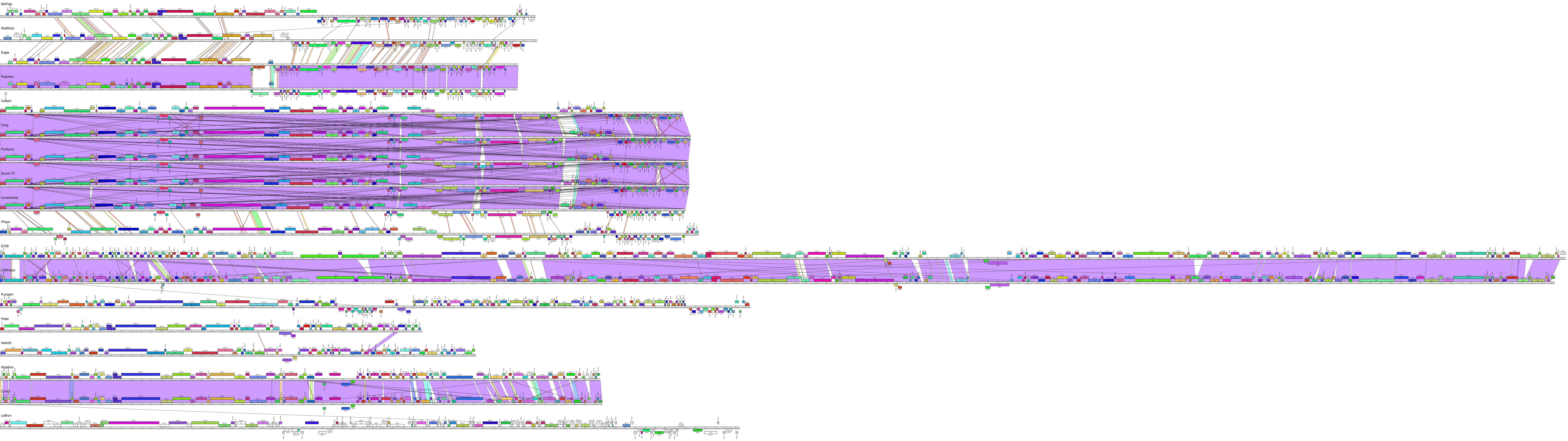
SkiPole
RedRock
Eagle
Peaches
Colbert
Fang
Puhltonio
Scoot17C
UncleHowie
Phlyer
ET08
LRRHood
Pumpkin
Hope
Island3
Angelica
CrimD
LeBron
